# Manufacture of highly loaded silica-supported cobalt Fischer–Tropsch catalysts from a metal organic framework

**DOI:** 10.1038/s41467-017-01910-9

**Published:** 2017-11-22

**Authors:** Xiaohui Sun, Alma I. Olivos Suarez, Mark Meijerink, Tom van Deelen, Samy Ould-Chikh, Jovana Zečević, Krijn P. de Jong, Freek Kapteijn, Jorge Gascon

**Affiliations:** 1 Catalysis Engineering, Chemical Engineering Department, Delft University of Technology, Van der Maasweg 9, 2629 HZ Delft, The Netherlands; 2 Inorganic Chemistry and Catalysis, Debye Institute for Nanomaterials Science, Utrecht University, Universiteitsweg 99, 3584 CG Utrecht, The Netherlands; 3 King Abdullah University of Science and Technology, KAUST Catalysis Center, Advanced Catalytic Materials, Thuwal, 23955 Saudi Arabia

## Abstract

The development of synthetic protocols for the preparation of highly loaded metal nanoparticle-supported catalysts has received a great deal of attention over the last few decades. Independently controlling metal loading, nanoparticle size, distribution, and accessibility has proven challenging because of the clear interdependence between these crucial performance parameters. Here we present a stepwise methodology that, making use of a cobalt-containing metal organic framework as hard template (ZIF-67), allows addressing this long-standing challenge. Condensation of silica in the Co-metal organic framework pore space followed by pyrolysis and subsequent calcination of these composites renders highly loaded cobalt nanocomposites (~ 50 wt.% Co), with cobalt oxide reducibility in the order of 80% and a good particle dispersion, that exhibit high activity, C5 + selectivity and stability in Fischer–Tropsch synthesis.

## Abstract (web summary)

Preparation of supported catalysts with high nanoparticle loading is a considerable synthetic challenge. Here, by using a metal organic framework as sacrificial template, the authors report a cobalt catalyst with a 50% Co loading with superior activity in the C5+ selective production of hydrocarbons from syngas.

## Introduction

Metal (oxide) nanoparticles are instrumental in the development of new applications: from the production of fuels and chemicals through catalytic processes^1^ to nanoelectronics^2^ and energy conversion and storage^3^. Because most chemical and electronic phenomena occur at the surface, the intrinsic properties of nanoparticles depend strongly on their size, spatial distribution and even on their shape^4, 5^. In general, small nanoparticles show high surface energies and are thermally unstable and prone to aggregate into larger clusters^6^. To tackle this issue, a general strategy consists of the use of supports with high surface area and well-developed porosity (e.g., SiO_2_ and Al_2_O_3_) that stabilize and prevent nanoparticle aggregation^5, 7, 8^.

Ion-adsorption^9, 10^, impregnation and subsequent drying^7^, or deposition–precipitation^11, 12^ are among the most commonly used methods for the preparation of supported nanoparticles. Metal loading, nanoparticle size, and distribution are the three most important parameters that define performance of supported nanoparticles. Although it would be ideal to control independently each one of these parameters, in reality a strong interdependence exists. For example, for the methods described above, metal loading and particle size usually go hand in hand as a result of the fact that bigger nanoparticles and/or clusters are formed when high metal loadings are used. This interdependence is a clear drawback for the development of more efficient nanoparticle based composites for application in, i.e., heterogeneous catalysis. Structure sensitive reactions such as Fischer–Tropsch synthesis (FTS) are an outstanding example. For this specific process, when Co is used as the active metal, catalytic activity and selectivity to long-chain hydrocarbons are maximized when nanoparticles in the order of 8–30 nm are used^13–15^. Because of this reason, impregnation is the most widely used method for the preparation of industrial FTS catalysts^16, 17^. However, using this method the maximum metal loading usually achieved is not higher than a 20 wt.%^7, 18, 19^. As an alternative, deposition–precipitation methods have been developed to achieve higher metal loadings^20, 21^. Yet, a large fraction of irreducible species (i.e., metal silicates and/or aluminates) is formed, resulting in non-optimal utilization of the active phase (that requires to be in the metallic form under reaction conditions)^21, 22^. In this respect, it is not surprising that the development of alternative methods for the preparation of these composites is gaining a tremendous attention in both the open and patent literature.

Among the different strategies suggested in literature, the use of metal organic-frameworks (MOFs) as precursors for the synthesis of nanomaterials such as metal (oxide) nanoparticles^23–26^, porous silica^27, 28^, or nanoporous carbons^29^ offers unrivaled design possibilities, as we also demonstrate in this work. Herein we report a multi-step approach for the preparation of highly loaded Co on silica FTS catalysts that circumvents the interdependence between metal loading, active site dispersion, and accessibility. By using this approach, highly loaded cobalt nanocomposites (~ 50 wt.% Co) with cobalt oxide reducibility in the order of 80% and good particle dispersion were synthesized and tested in FTS. These catalysts exhibit high activity, C5 + selectivity, and excellent stability.

## Results

### Catalyst synthesis and characterization

**Fig. 1:**
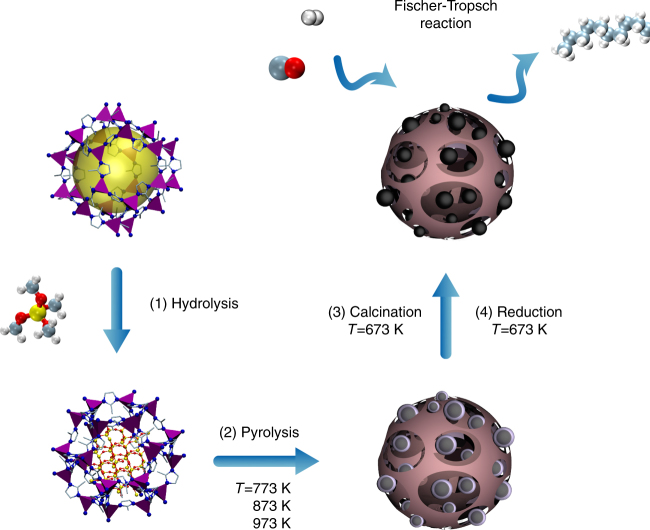
Schematic illustration of the synthesis of the Co@SiO_2_ catalysts. (**1**) Impregnation and hydrolysis of TMOS molecules in the porosity of ZIF-67. (**2**) Pyrolysis of the mixture of ZIF-67@SiO_2_ in N_2_ to decompose ZIF-67 and form Co@C-SiO_2_. (**3**) Calcination of the Co@C-SiO_2_ in air leads to carbon removal and oxidation of Co. (**4**) Reduction of the Co@SiO_2_ in H_2_ leads to the formation of metallic Co for Fischer–Tropsch synthesis. The resulting composite is an excellent catalyst for the low temperature Fischer–Tropsch synthesis

Figure 1 illustrates the followed synthetic procedure. We used the zeolitic imidazolate-framework ZIF-67, containing a 30 wt.% Co (Co(MeIm)_2_, MeIm = 2-methylimidazolate) and tetramethyl orthosilicate (TMOS) as starting materials for the synthesis of cobalt catalysts. In this approach, a TMOS impregnated ZIF-67 was first subjected to a wet N_2_ flow under ambient conditions to facilitate TMOS hydrolysis inside the pores of the MOF. The obtained ZIF-67@SiO_2_ sample was then pyrolyzed at different temperatures in the range of 773–973 K under N_2_ for 4 h, followed by calcination in air at 673 K for 2 h. The catalysts after pyrolysis and calcination are denoted as Co@C-SiO_2_-*T* and Co@SiO_2_-*T*, respectively, with *T* representing the pyrolysis temperature. For comparison, a Co@SiO2-*cal*. sample was also prepared by direct calcination (skipping the intermediate pyrolysis step) of ZIF-67@SiO_2_ in air (details of the preparation process for all materials are shown in the Methods section). The Co loadings of the Co@SiO_2_ catalysts are ~50 wt.% (Supplementary Table 1).

The X-ray diffraction (XRD) pattern of the original ZIF-67 (Supplementary Fig. 1), confirms the structure of the MOF precursor^30^. Thermogravimetric (TG) analysis in N_2_ atmosphere of the hydrolyzed ZIF-67@SiO_2_ indicates that the complete pyrolytic decomposition of the crystalline ZIF-67 occurs in the range of 800–850 K (Supplementary Fig. 2), further confirmed by XRD (Supplementary Fig. 3a). After the pyrolysis step, graphite (2*Φ* = 30.6^°^) and metallic cobalt (2*Φ* = 51.8^°^, 60.6^°^) phases are formed. Notably, when a higher pyrolysis temperature is used, these peaks become much narrower and sharper, indicating a higher graphitization degree and a larger crystallite size of cobalt nanoparticles^31^. After the additional calcination step, the characteristic peaks corresponding to ZIF-67, graphite, and metallic cobalt phases have disappeared and only the Co_3_O_4_ phase is observed (Supplementary Fig. 3b).

Both ZIF-67 and ZIF-67@SiO_2_ display type-I N_2_ sorption isotherm (Supplementary Fig. 4a) typically associated with microporosity^32^. The Brunauer-Emmett-Teller area (*S*_BET_) and pore volume (*V*_p_) decreases from 1930 m^2^ g^−1^ and 0.71 cm^3^ g^−1^ to 1430 m^2^ g^−1^ and 0.56 cm^3^ g^−1^ after incorporation of SiO_2_ (Supplementary Table 1)^33^. In contrast with the original ZIF-67@SiO_2_, the *S*_BET_ and *V*_*p*_ of all Co@SiO_2_ catalysts decreases drastically and exhibit type IV isotherms with type H_3_ hysteresis that closes at *P*/*P*_*0*_ ≈ 0.4, suggesting the presence of a predominantly mesoporous structure which is the result of the agglomeration of small SiO_2_ particles (Supplementary Fig. 4b).

**Fig. 2:**
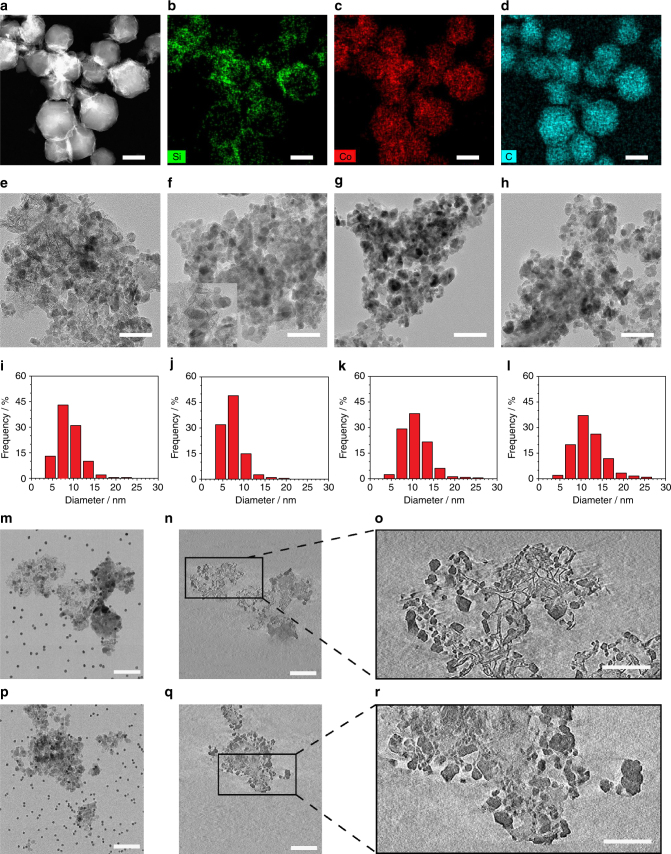
Electron microscopy images and corresponding nanoparticle size distributions of cobalt based samples. **a** High-angle annular dark-field scanning electron (HAADF-STEM) micrograph of ZIF-67@SiO_2_ (scale bar 200 nm). Elemental mapping of **b** Si, **c** Co, and **d** C in ZIF-67@SiO_2_ sample (scale bars 200 nm). TEM micrograph of **e** Co@SiO_2_-*cal*, **f** Co@SiO_2_-*773* with an inset of the observable needle-like structure, **g** Co@SiO_2_-*873* and **h** Co@SiO_2_-*973* (scale bars from (e) – (h) 50 nm). Particle size histograms obtained from TEM analysis for **i** Co@SiO_2_-*cal*, **j** Co@SiO_2_-*773*, **k** Co@SiO_2_-*873*, and **l** Co@SiO2-*973*. Electron tomography results for **m**, **n**, **o** Co@SiO_2_-*cal* (scale bar 50, 50, and 100 nm, respectively), and **p**, **q**, **r** Co@SiO_2_-*873* (scale bar 50, 50, and 100 nm, respectively)

**Fig. 3:**
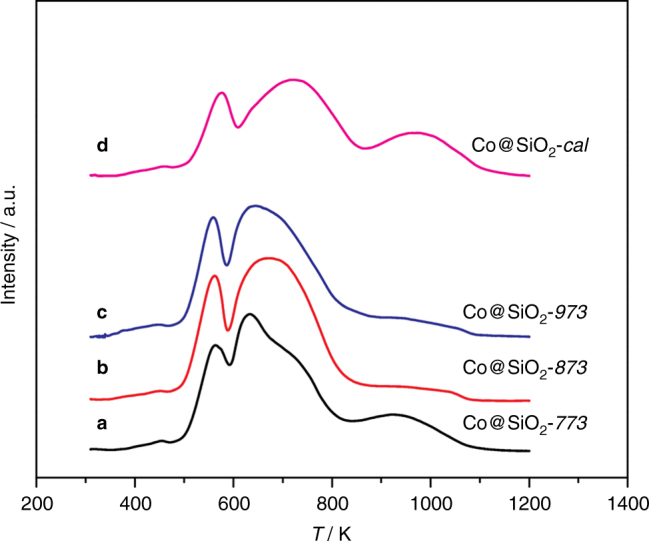
TPR(H_2_) profiles of Co@SiO_2_ catalysts. **a** Co@SiO_2_-*773*, **b** Co@SiO_2_-*873*, **c** Co@SiO_2_-*973*, and **d** Co@SiO2-*cal*. The TPR(H_2_) experiments were performed from 303 to 1223 K at a ramp of 5 K min^−1^ in 10 vol.% H_2_/Ar

Transmission electron microscopy (TEM) and high-resolution transmission electron microscopy (HR-TEM) analysis in combination with elemental mapping (STEM/EDX (elemental energy dispersive X-ray)) give further information on the textural properties of the composites at different synthesis stages. High-angle annular dark-field scanning electron (HAADF-STEM) (Fig. 2a) analysis shows a well-defined rhombic dodecahedral morphology (~ 250 nm) of the ZIF-67@SiO_2_ catalysts similar to that of the original ZIF-67^30^, whereas elemental mapping demonstrates an homogeneous dispersion of Si, Co and C (Fig. 2b–d). After pyrolysis under N_2_ atmosphere, well dispersed cobalt nanoparticles in the carbon matrix can be observed in Co@C-SiO_2_-*T* samples (Supplementary Fig. 5a–c), with average particle size increasing from 5.4 nm in Co@C-SiO_2_-*773* to 11.0 nm in Co@C-SiO_2_-*873*, and 13.3 nm in Co@C-SiO_2_-*973* (Supplementary Fig. 5d–f). According to HR-TEM, during pyrolysis, cobalt nanoparticles are encapsulated by multilayers of graphitic-carbon shells (Supplementary Fig. 5g–i) that render them, most likely, inaccessible. XRD analysis further confirm this observation, since reoxidation of most Co does not occur upon exposure to atmospheric conditions (*vide supra*). In addition, leaching experiment using HCl demonstrates that only a 30% of cobalt can be leached (Supplementary Table 2). The subsequent calcination removes the graphite shells and oxidizes metallic cobalt to Co_3_O_4_ (Fig. 3f–h, and Supplementary Fig. 6b–d), but hardly affects Co-particle size (Fig. 3j–l, and Table 1). No large cobalt clusters can be found in the Co@SiO_2_-*873* sample even after reduction in H_2_ at 673 K for 10 h (Supplementary Fig. 7a–d). Interestingly, Co@SiO_2_-*cal*. (Fig. 2e, m and Supplementary Fig. 8a) and Co@SiO_2_-*773* (the inset of Fig. 2f) show the presence of needle-like structures absent in samples pyrolyzed at higher temperatures. Additional analysis by combining TEM and EDX (Supplementary Fig. 8b, c) reveals the presence of both Si and Co in needle- rich areas and made us tentatively attribute this morphology to the formation of cobalt phyllosilicates^34^.

**Table 1:** Average cobalt particle size and DOR of Co@SiO_2_ catalysts

Samples	*d*_Co_^a^	*d*_Co_^b^	DOR (%)
Co@SiO_2_-*773*	8.6	7.6	66
Co@SiO_2_-*873*	12.3	11.8	78
Co@SiO_2_-*973*	14.3	13.5	79
Co/SiO_2_-*cal*.	10.7	9.5	52

^a^Cobalt particle size is obtained from TEM analysis using at least 200 Co_3_O_4_ nanoparticles and calculated from Co_3_O_4_ particle size using Co and Co_3_O_4_ densities ^b^ Cobalt particle size is calculated from H_2_-chemisorption assuming the surface stoichiometry H/Co = 1 and an atomic cross-sectional area of 0.0662 nm^2^. Cobalt oxide degree of reduction (DOR)

The reducibility of the metallic species in all calcined samples was studied by temperature-programmed reduction in H_2_ (TPR(H_2_)). All of the Co@SiO_2_ samples exhibit two overlapping reduction peaks centered at ~ 570 K and 700 K, and a broad reduction band between 850 and 1150 K, as shown in Fig. 3. The first two peaks are ascribed to the two-step reduction of Co_3_O_4_ via CoO to metallic Co^35^, along with gasification of the residual carbon in the samples (Supplementary Fig. 9), whereas the broad feature illustrates the reduction of highly dispersed cobalt species in strong interaction with the SiO_2_ support (e.g., cobalt phyllosilicate)^36^. In the case of Co@SiO_2_-*cal*., the second reduction occurs at a slightly higher temperature, indicative of a stronger interaction between cobalt nanoparticles and support, most likely due to the presence of very small cobalt particles, as proven from the electron tomography results in Fig. 2m. Moreover, the broad high-temperature band in Co@SiO2-*cal*. and Co@SiO_2_-*773* implies the presence of a large fraction of irreducible cobalt silicates, in agreement with the TEM analysis above. This is further confirmed by the lower degree of reduction (DOR) of cobalt oxide in Co@SiO_2_-*cal*. (52%) and Co@SiO_2_-*773* (66%) than in Co@SiO_2_-*873* (78%) and Co@SiO_2_-*973* (79%), see Table 1. These results highlight the importance of the intermediate pyrolysis step at a sufficiently high temperature as to achieve full destruction of the ZIF-67 sample to prevent the formation of irreducible cobalt silicate and therefore ensure an almost full utilization of the catalyst’s cobalt loading.

### Catalytic results

**Fig. 4:**
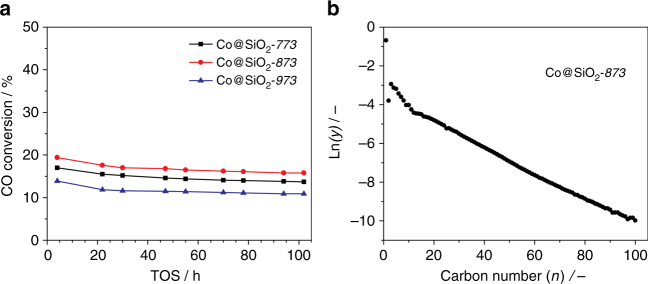
Catalytic performance. **a** Time-on-stream evolution of CO conversion for the Co@SiO_2_ catalysts. **b** Molar fraction distribution of FTS products from Co@SiO_2_-*873* after 201 h on stream. Chain growth probability (*α* = 0.94) obtained from the ASF plot in the C15-C100 hydrocarbon range. Reaction conditions: 483 K, 20 bar, and H_2_/CO = 1, and syngas flow of 40 ml min^−1^

The Co@SiO_2_ catalysts were tested in the FTS at 483 K, 20 bar, H_2_/CO = 1, and a space velocity of 0.5 mol_CO_ g^−1^_cat._ h^−1^. Figure 4a shows time-on-stream (TOS) evolution of CO conversion. All catalysts exhibit a good stability, and differences observed in activity are in line with the observed textural properties. Co@SiO_2_-*873* displays the highest CO conversion, followed by Co@SiO_2_-*773* and Co@SiO_2_-*973*. Table 2 summarizes cobalt-time-yield (CTY), apparent turnover frequencies (TOF) and product selectivity for these catalysts after 102 h on stream. When CTY is plotted as a function of the pyrolysis temperature, a volcano-like curve is obtained, with an optimum for the sample pyrolyzed at 873 K. The TOF values calculated for samples pyrolyzed at 873 and 973 K are similar and higher than that of the Co@SiO_2_-*773* sample. The FTS process occurs on the surface of metallic cobalt nanoparticles with an optimal particle size around 10 nm. On one hand, small cobalt nanoparticles normally possess a large fraction of low-coordinated surface sites (i.e., corner, kink, edge etc.), which to a large extent hamper CO dissociation and/or CH_x_ hydrogenation^13, 37^. Hence, we attribute the superior activity of Co@SiO_2_-*873* to the high Co reducibility and the optimal Co-particle size (Table 1)^13, 14, 16, 38, 39^. On the other hand, small cobalt nanoparticles have only few step sites, known for C–C formation towards long chain hydrocarbons, therefore resulting in a high methane selectivity^40, 41^. Thus, the larger Co-particle size in the Co@SiO_2_-*873* and Co@SiO_2_-*973* samples when compared to Co@SiO_2_-*773* results in a lower CH_4_ and a higher C5 + selectivity for these catalysts (Table 2), in excellent agreement with literature^13^. We argue that the low H_2_/CO ratio and operating temperature applied in this work (H_2_/CO = 1,483 K) along with an optimal cobalt particle size in the synthesized Co@SiO_2_-*873* catalyst result in a chain growth probability (*α*) as high as 0.94^42, 43^.

**Table 2:** Catalytic performance of Co@SiO_2_ catalysts after 102 h TOS

Sample	Sample weight (mg)	Cobalt loading (wt.%)	*X*_CO_ (%)	CTY (10^−5^ mol_CO_g^−1^_Co_s^−1^)	TOF (10^−2^ s^−1^)	*S* (%)		
						C1	C2–C4	C5+
Co@SiO_2_-*773*	100	49	13.7	4.0	1.9	6.5	6.3	87.2
Co@SiO_2_-*873*	100	51	15.8	4.4	3.1	5.3	4.2	90.5
Co@SiO_2_-*973*	100	50	10.9	3.3	2.8	5.8	4.7	89.5
Co/SiO_2_-*cal*.	100	46	10.6	3.3	1.9	7.5	6.8	85.7

Carbon conversion (*X*, %), activity per gram of Co (CTY), apparent turnover frequency (TOF, mol CO converted per mol Co surface atoms per second), hydrocarbon selectivity (*S*, %). FTS experiments were carried out at 483 K, 20 bar, and H_2_/CO = 1, and syngas flow of 40 ml min^−1^

The performance of the Co@SiO_2_-*cal*. sample further emphasizes the key role of the intermediate pyrolysis step (Table 2). A high initial CO conversion over this sample along with a clear deactivation during the first 50 h on stream (Supplementary Fig. 10) is observed. We attribute the severe deactivation at the initial stage to the presence of a substantial amount of small cobalt nanoparticles (<4 nm), that are more susceptible to aggregation and/or oxidation than larger particles during high-pressure FTS and which also more selective for the formation of CH_4_^44, 45^_._ In addition, although pyrolysis of Co-based MOFs under an inert atmosphere has recently been demonstrated as a promising route to prepare highly loaded Co@C hybrids with controllable cobalt particle size and distribution^25, 46–49^, these directly pyrolyzed samples such as Co@C-*873* and Co@C-SiO_2_-*873* synthesized in this work show a poor activity and low C5 + selectivity along with an unacceptable CH_4_ selectivity in the FTS process under the same conditions as Co@SiO_2_ catalysts (Supplementary Fig. 11 and Supplementary Table 3). The inferior performance of these pyrolyzed samples can be ascribed to the inaccessibility of most cobalt nanoparticles, which are completely encapsulated by graphitic shells. (Supplementary Fig. 12 and Supplementary Table 2)^50, 51^. Comparison of our results demonstrates the importance of the synthetic protocol here presented).

## Discussion

The results here presented demonstrate that the stepwise hydrolysis-pyrolysis-calcination methodology is a promising route to synthesize highly loaded Co@SiO_2_ catalysts using ZIF-67 as a sacrificial template and TMOS as silicon source. During the high-temperature pyrolysis, the ZIF-67 structure decomposes, generating cobalt nanoparticles encapsulated by graphitic-carbon shells, which prevent the formation of large agglomerates, controlling in this way cobalt particle dispersity, whereas optimization of the pyrolysis temperature improves cobalt reducibility.

**Fig. 5:**
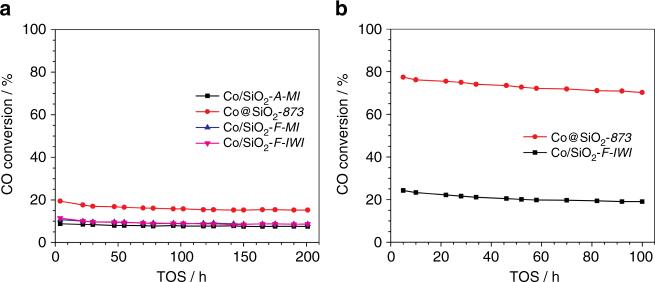
Catalytic performance. **a** Time-on-stream evolution of CO conversion for the Co@SiO_2_-*873* and Co/SiO_2_ catalysts prepared using conventional methods. *M*’ refers to melt infiltration. *IWI* refers to incipient wetness impregnation. *A* refers to Aerosil-200 support and *F* refers to CARiACT Q-10 support. Reaction conditions: 483 K, 20 bar, H_2_/CO = 1, and syngas flow of 40 ml min^−1^. **b** Time-on-stream evolution of CO conversion for the Co@SiO_2_-*873* and Co/SiO_2_ *-F-TIWI* catalysts prepared using two-step incipient wetness impregnation method (*TIWI*). Reaction conditions: 483 K, 26 bar, H_2_/CO = 2, and syngas flow of 40 ml min^−1^

To further demonstrate the advantages of this synthetic methodology, we prepared additional highly loaded Co catalysts, with Co supported on commercially available Aerosil-200 (denoted as A) or CARiACT Q-10 (denoted as F) silica, by using melt infiltration (MI). Also two benchmark Co/SiO_2_ catalysts with cobalt loading of 16 wt.% and 32 wt.%, respectively, were prepared by means of incipient wetness impregnation (IWI). The 32 wt.%Co/SiO_2_ *-F-TIWI* and 40 wt.%Co/SiO_2_ *-A-MI* catalysts consist mostly of large aggregates (Supplementary Fig. 13a–c, and Supplementary Fig. 14a, b) as a result of the lower versatility of the MI and IWI methods for high cobalt loadings. The comparison between the FTS performance of these catalysts and Co@SiO_2_-*873* is shown in Fig. 5a, b and Table 3. Under the studied conditions, the Co@SiO_2_-*873* displays a CTY at least 1.5 times (H_2_/CO = 1) (entry 1–4, Table 3) and/or 2.2 times (H_2_/CO = 2) (entry 5 and 7, Table 3) higher than the other samples (in spite of the higher Co loading) and a comparable C5 + selectivity (~ 83%) to its Co/SiO_2_ *-F-TIWI* counterpart at a similar CO conversion level (~ 26%). Interestingly, TEM images of the Co@SiO_2_-*873* catalyst after 201 h TOS show a very good dispersion of cobalt nanoparticles on the SiO_2_ support along with very few aggregates (Supplementary Fig. 15a–d), in good agreement with the observed very mild catalyst deactivation with time-on stream. In comparison with other highly loaded catalysts prepared using traditional methods, the optimal particle size and high stability of cobalt nanoparticles in Co@SiO_2_-*873* lead to more available cobalt sites and explain the high activity of Co@SiO_2_-*873* in the FTS process^7^.

**Table 3:** Catalytic performance of Co@SiO_2_-*873* and Co/SiO_2_ catalysts prepared using conventional methods

Sample	Sample weight (mg)	Cobalt loading (wt.%)	*X*_CO_ (%)	CTY (10^−5^ mol_CO_g^−1^_Co_s^−1^)	*S* (%)			
					C1	C2–C4	C5+	CO2
Co@SiO_2_-873^a^	100	51	15.2	4.2	5.2	3.8	91.0	—
Co/SiO_2_-*A-MI* ^a^	100	42	7.5	2.6	4.5	4.1	91.5	—
Co/SiO_2_-*F-MI* ^a^	100	42	8.6	3.0	4.8	4.3	90.9	—
Co/SiO_2_-F-IWI^a^	250	16.5	8.7	3.1	4.7	4.9	90.4	—
Co@SiO_2_-*873* ^b^	175	51	70.2	7.8	9.7	5.2	84.7	0.4
			25.8	6.4	10.7	6.5	82.8^c^	—
Co/SiO_2_-*F-TIWI* ^b^	175	32	19.1	3.5	9.0	7.4	83.6	—
			26.0	3.4	9.3	7.6	83.0^c^	—

Carbon conversion (*X*_CO_, %), activity per gram of Co (CTY), hydrocarbon selectivity (*S*, %). ^a^ FTS experiments were carried out at 483 K, 20 bar, and H_2_/CO = 1, and syngas flow of 40 ml min^−1^, and data were collected after 201 h TOS; ^b^ FTS experiments were carried out at 483 K, 26 bar, and H_2_/CO = 2, and syngas flow of 40 ml min^−1^, and data were collected after 100 h TOS. ^c^ C5 + selectivity was obtained after 118 h TOS by changing the feed flow rate after 100 h TOS

Overall, our results further highlight the potential and versatility of the use of MOFs as catalyst templates and opens the door to the controlled fabrication of highly loaded, accessible, active and stable metal supported catalysts thus coping with a major challenge in materials science and industrial catalysis.

## Methods

### Synthesis of the parent ZIF-67

In the synthesis of ZIF-67, 2.933 g of Co(NO_3_)_2_·6H_2_O and 6.489 g of 2-methylimidazole (MeIm) were separately dissolved in 200 ml methanol. The latter clear solution was rapidly poured into the former pink solution with vigorous stirring for 24 h at room temperature. Afterwards, the bright purple products were collected by filtration, washed with methanol, and dried at 353 K for 10 h under vacuum.

### Synthesis of ZIF-67@SiO_2_

A total of 0.8 g of the synthesized ZIF-67 was immersed in 5 ml TMOS in an autoclave, which was further transferred into a rotation oven and heated up to 333 K overnight. After the oven was cooled down to room temperature, the mixture was carefully washed with 1 ml ethanol to remove the excess TMOS on the external surface of ZIF-67 by filtration. Then the purple material was placed in a cotton thimble of 22 mm diameter and placed in a glass tube of 25 mm diameter. The glass tube was fitted to a round bottom flask containing 500 ml of water. A needle to bubble the water with 10 ml min^−1^ of N_2_ flux was also fitted. The temperature was raised to 323 K to create a wet N_2_ stream to directly hydrolyze the TMOS molecules for 30 h, followed by air drying at 333 K and vacuum drying at 373 K for 10 h, successively. The obtained sample was denoted as ZIF-67@SiO_2_.

### Synthesis of Co@C-SiO_2_-*T*

A total of 0.8 g of ZIF-67@SiO_2_ were transferred into a quartz tubular reactor (~*L* = 1.0 m x ID = 5.0 cm) horizontally situated in a ceramic fiber oven (Carbolite, Sheffield). The reactor was flushed with N_2_ at 303 K for 0.5 h, followed by direct carbonization at different temperature for 4 h under N_2_ (150 ml min^−1^) at a ramp of 2 K min^−1^. The obtained sample was denoted as Co@C-SiO_2_-*T*, where *T* (*T* = 773, 873, 973 K) refers to the pyrolysis temperature.

### Synthesis of Co@SiO_2_-*T* and Co@SiO_2_-*cal*

The obtained Co@C-SiO_2_-*T* samples were further calcined at 673 K in air (150 ml min^−1^) for 2 h at a ramp of 1 K min^−1^, and denoted as Co@SiO_2_-*T*, where *T* (*T* = 773, 873, 973 K) refers to the pyrolysis temperature. For comparison, 0.8 g of ZIF-67@SiO_2_ was directly calcined at 673 K in air (150 ml min^−1^) for 2 h at a ramp of 1 K min^−1^, and this sample was denoted as Co@SiO_2_-*cal*.

### Synthesis of Co@C-*873*

Co@C-*873* was prepared by pyrolysis of 0.8 g ZIF-67 at 873 K for 4 h under 150 ml min^−1^ N_2_ flow at a ramp of 2 K min^−1^.

### Synthesis of Co/SiO_2_ catalysts with conventional methods

For the MI samples, 2.9 g Co(NO_3_)_2_·6H_2_O and 0.6 g of degassed SiO_2_ support (Aerosil-200 or CARiACT Q-10) were physically mixed in a mortar with a pestle under ambient conditions until the powder was homogeneously pink. Then the samples were transferred into a Teflon-lined steel autoclave and kept at 333 K for 24 h, followed by calcination by heating to 673 K (1 K min^−1^, 2 h) in a flow of air (150 ml min^−1^ for 0.8 g precursor loaded catalyst) in the same setup as mentioned above. The obtained samples were denoted as Co/SiO_2_-*A-MI* (Aerosil-200) and Co/SiO_2_-*F-MI* (CARiACT Q-10), respectively. For the IWI sample, 1 g of degassed SiO_2_ support (CARiACT Q-10) was impregnated with 1 ml of aqueous cobalt nitrate solution. The catalyst precursor was dried overnight under vacuum at 373 K followed by calcination by heating to 673 K (1 K min^−1^, 2 h) in a flow of air (150 ml min^−1^ for 0.8 g precursor loaded catalyst) in the same setup as mentioned above. The obtained sample was denoted as Co/SiO_2_-*F-IWI*. Co/SiO_2_-*F-TIWI* sample was prepared by two-step IWI of Co(NO_3_)_2_·6H_2_O aqueous solution to SiO_2_ support (CARiACT Q-10), followed by drying overnight under vacuum at 373 K, and calcination by heating to 673 K (1 K min^−1^, 2 h) in a flow of air (150 ml min^−1^ for 0.8 g precursor loaded catalyst) in the same setup as mentioned above.

### Characterization

The Co contents in the samples were measured by atomic adsorption spectroscopy (AAnalyst 200, Perkin Elmer, USA). PXRD patterns were measured by a Bruker D8 Advance X-ray diffractometer using monochromatic Co *Kα* radiation (*λ* = 0.179026 nm). N_2_ adsorption-desorption isotherms were obtained using a Micromeritics Tristar 3020 at 77 K, and samples were outgassed under vacuum at 423 K overnight prior to the analysis. For the analysis, the BET area was determined as outlined in Lange et al.^33^. The mesopore surface area was obtained from the *t*-plot applied to the N_2_ isotherm. TG analysis was carried out using a Mettler Toledo TGA/SDTA851e instrument by heating samples in N_2_ (100 ml min^−1^) from room temperature to 1073 K at a ramp rate of 5 K min^−1^. TEM imaging and EDX mapping were performed on a JEM-2100 (JEOL) and a Talos F200X (FEI) microscopes operated at 200 kV. Tilt series of bright-field TEM images for electron tomography were taken with a Talos F200X (FEI) microscope, over the angle range of ± 76° with a tilt increment of 2°. Tilt series were aligned and reconstructed using IMOD software package^52^. Cobalt particle diameter (*d*_TEM_) was calculated based on a minimum of 200 nanoparticles using the equation (1)

1 $$d_{TEM} = {\sum} {_in_id_i^3} /{\sum} {_in_id_i^2}$$

where *n*_*i*_ is the number of particles with diameter of *d*_*i*_. The bright-field and HAADF-STEM imaging of the Co/SiO_2_ *-F-TIWI* and spent Co@SiO_2_-*873* catalysts were performed using a FEI TEM (model Titan 80–300 ST) at 300 kV. Temperature-programmed reduction in hydrogen (TPR(H_2_)) was performed in a flow of 10 vol.% H_2_/Ar (30 ml min^−1^) at a heating rate of 5 K min^−1^ from ambient temperature to 1223 K. The DOR was measured using TGA (Mettler Toledo TGA/SDTA851e) in a flow of 10% H_2_/He. The samples were heated to 673 K and held there for 8 h (No weight loss was obtained after this time). After that the temperature was further increased to 1273 K (5 K min^−1^). The DOR of cobalt was calculated using the equation (2)

2 $$\left( {n_{Co}^{total} - n_{Co}^{  >673}} \right)/n_{Co}^{total}$$

A Micromeritics ASAP 2020 was used to measure H_2_-chemisorption. Samples dried at 100 °C were submitted to reduction in H_2_ at 673 K (10 h, 5 K min^−1^) and evacuation at the same temperature. Isotherms were measured at 423 K. The accesible cobalt surface areas were calculated assuming a one to one stoichiometry (H:Co) and a Co-atomic cross section of 0.0662 nm^2^.

### Catalytic testing

The FTS was carried out in a parallel 6-flow fixed-bed microreactor setup as previously described^53^. Certain amount of catalyst was mixed with SiC of similar size and loaded into a stainless steel tube lined with a quartz layer. Catalysts were reduced in situ in pure H_2_ at 673 K for 10 h at 2 K min^−1^. Afterwards, the reactors were cooled to 453 K at which the pressure was increased to the target pressure (20 or 26 bar) under H_2_. Then, a CO flow was gradually introduced into the system, and finally reached an H_2_/CO ratio of 1 or 2 with syngas flow of 40 ml min^−1^. Next, the temperature was increased to the reaction temperature of 483 K at 2 K min^−1^. The C5 + selectivity was calculated from the CO conversion by subtracting the fraction of CO used for the formation of C_1_ to C_4_ products, as determined via online GC (Hewlett Packard 5890, Series II) using N_2_ as an internal standard, from the total amount of CO converted.

### Data availability

The authors declare that all other relevant data not included in the Supplementary Information and supporting the findings of this study are available on request.

## Electronic supplementary material

Peer Review File

Supplementary Information
