# Supplementary material for: Manufacture of highly loaded silica-supported cobalt Fischer–Tropsch catalysts from a metal organic framework
Source: Nat Commun. 2017 Nov 22;8:1680. doi: 10.1038/s41467-017-01910-9 (PMC5698480; doi:10.1038/s41467-017-01910-9)
Supplement: Supplementary file 2 — Supplementary Information [file 41467_2017_1910_MOESM2_ESM.pdf]

Supplementary Tables

Supplementary Table 1 Textural properties of Co-based catalysts obtained from N_2 adsorption isotherms at 77 K, and cobalt loading of Co containing catalysts.

Samples	S / (m^2/g)			V_p / (cm^3/g)			Co loading (wt. %)
	Total (S_{BET})	Micro	Meso[c]	Total	Micro	Meso	
ZIF-67[a]	1930	1910	20	0.71	0.68	0.03	30[d]
ZIF-67@SiO_2[a]	1430	1400	30	0.56	0.52	0.04	26[d]
Co@SiO_2-*773*[b]	260	35	225	0.46	0.02	0.44	49[e]
Co@SiO_2-*873*[b]	285	80	205	0.50	0.03	0.46	51[e]
Co@SiO_2-*973*[b]	305	70	235	0.51	0.03	0.48	50[e]
Co@SiO_2-*cal.*[b]	250	55	200	0.46	0.02	0.44	46[e]

a: Data obtained based on ZIF-67 support mass. b: Data obtained based on SiO_2 support mass. c: Mesopore surface area obtained from the *t*-plot applied to the N_2 isotherm. d: 10 mg samples were calcined in air (100 ml min^{-1}, STP) from 303 K to 1073 K at a heating rate of 5 K min^{-1}, and after 700 K no weight change was observed. Then Co loading was obtained based on Thermogravimetric (TG) analysis assuming that cobalt was fully converted to Co_3O_4. e: Data obtained based on atomic adsorption spectroscopy (AAS) analysis.

Supplementary Table 2 Cobalt loading in Co@C-*873* and Co@C-*873(al)* catalysts.

Sample	Cobalt loading (wt.%)
Co@C-*873*	32
Co@C-*873*(*al*)[a]	22

a: Co@C-*873(al)* was obtained by immersing 0.5 g Co@C-*873* in 500 ml of 0.5 M hydrochloric acid solution for 4 days at 303 K to dissolve the exposed cobalt nanoparticles, followed by washing with deionized water and drying at 323 K under vacuum.

Supplementary Table 3 Catalytic performance of Co/γ-Al_2O_3, Co@SiO_2-*873*, Co@C-*873*, and Co@C-SiO_2-*873* catalysts after 102 h *TOS*.

Sample	Sample weight (mg)	Cobalt loading (wt.%)	X_{CO} (%)	CTY (10^{-5} mol_{CO} g^{-1}_{Co} s^{-1})	*S* [%]		
					C1	C2-C4	C5+
Co/γ-Al_2O_3[a]	250	17	4.9	1.7	6.8	7.8	85.4
Co@SiO_2-*873*	100	51	15.8	4.4	5.3	4.2	90.5
Co@C-*873*	150	32	4.9	1.5	13.1	7.5	79.4
Co@C-SiO_2-*873*	150	28	5.8	2.1	17.1	11.1	71.7

a: Co/γ-Al_2O_3 was prepared using incipient wetness impregnation method with aqueous cobalt nitrate solution, followed by drying at 373 K under vacuum overnight and calcination under air flow (150 ml min^{-1}) for 2 h at a ramp rate of 1 K min^{-1}. Carbon conversion (X_{CO}, %), activity per gram of Co (*CTY*), hydrocarbon selectivity (*S*, %). FTS experiments were carried out at 483 K, 20 bar, and H_2/CO=1, and syngas flow of 40 ml min^{-1}.

Supplementary Figures

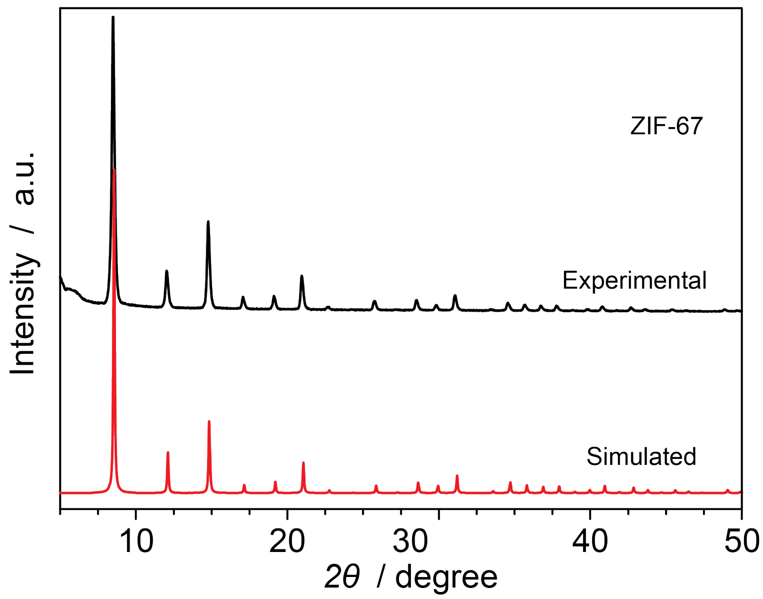

Supplementary Figure 1 X-ray diffraction patterns of the synthesized ZIF-67.

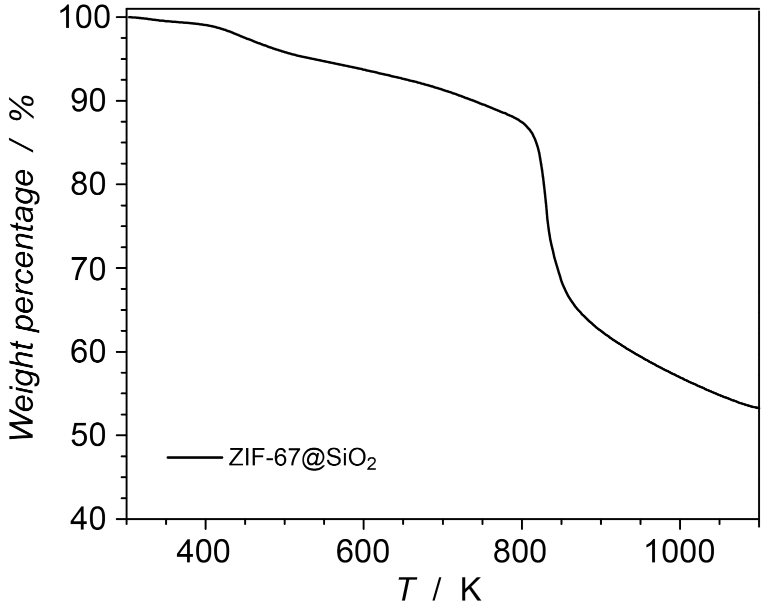

Supplementary Figure 2 TGA profile of ZIF-67@SiO_2 heated in N_2 flow (100 ml min^{-1} STP) at a ramp of 5 K min^{-1}. The complete decomposition of the ZIF-67 structure occurs between 800 and 850 K.

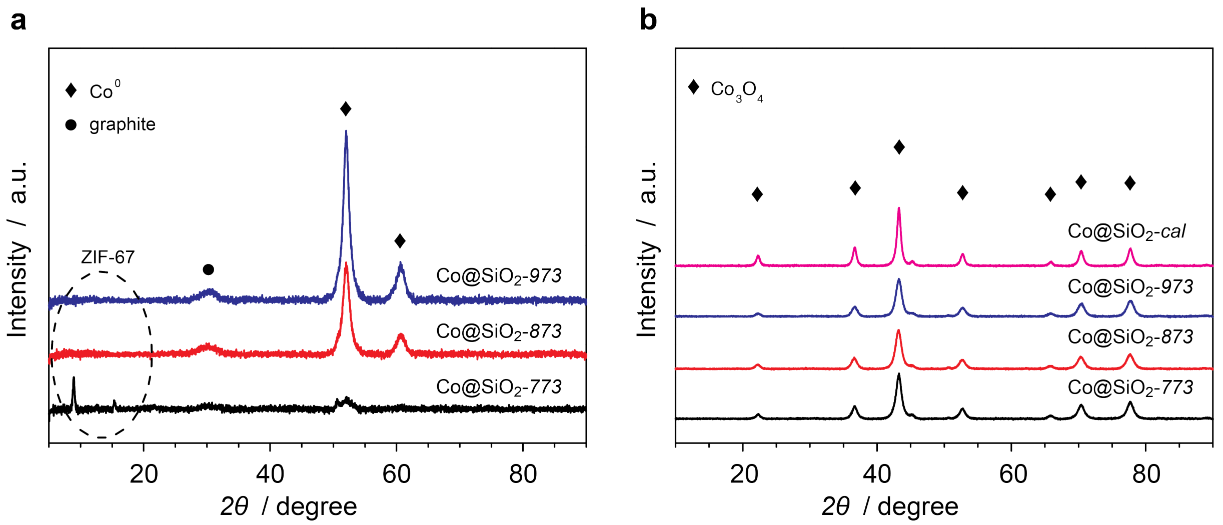

Supplementary Figure 3 X-ray diffraction patterns of the different catalyst samples. (a) Co@C-SiO_2 catalysts after pyrolysis at different temperatures under N_2 atmosphere. (b) Co@SiO_2 catalysts after calcination at 673 K for 2 h in air.

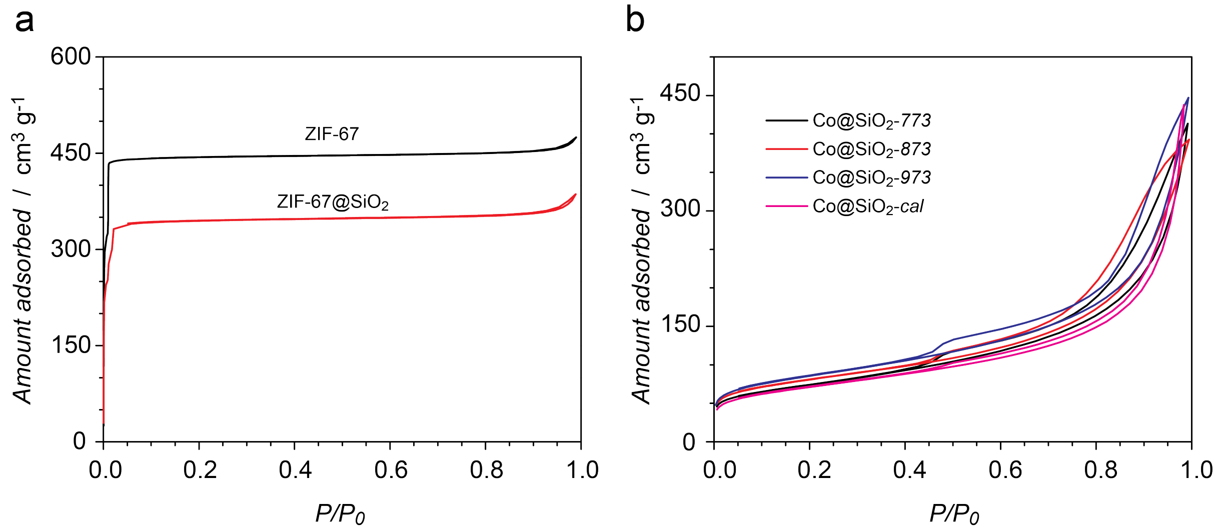

Supplementary Figure 4 N_2 adsorption-desorption isotherms (77 K) of different samples. (a) ZIF-67 and ZIF-67@SiO_2 samples after hydrolysis treatment, (b) Co@SiO_2 samples after calcination at 673 K for 2 h in air.

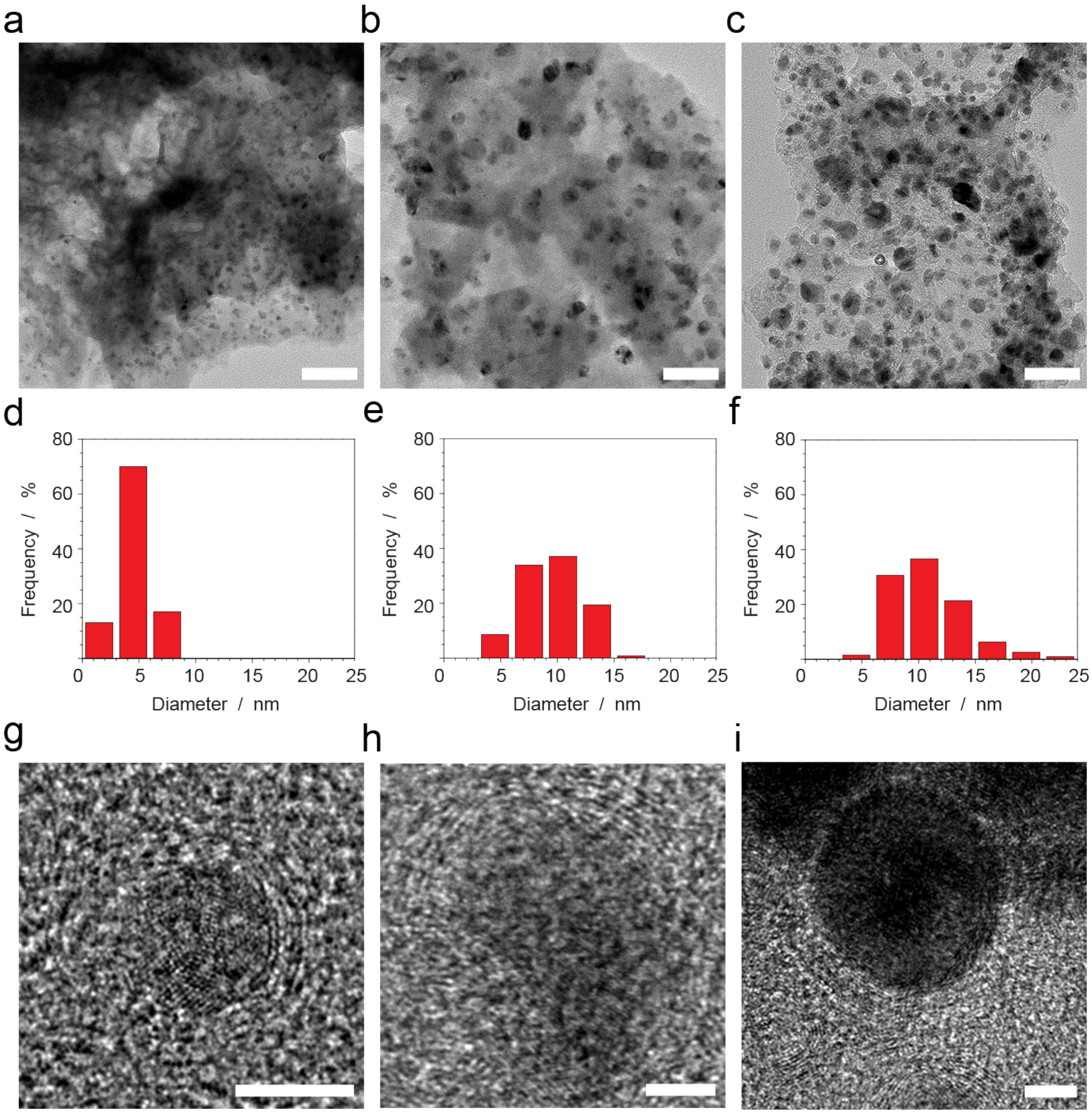

Supplementary Figure 5 TEM micrographs and corresponding nanoparticle size distributions of Co@C-SiO_2-*T* catalysts. (a) TEM micrograph of Co@C-SiO_2-*773* (scale bar 50 nm), (b) Co@C-SiO_2-*873* (scale bar 50 nm), and (c) Co@C-SiO_2-*973* (scale bar 50 nm). (d) Particle size histograms obtained from TEM analysis for Co@C-SiO_2-*773*, (e) Co@C-SiO_2-*873*, and (f) Co@C-SiO_2-*973*. (g) High-resolution TEM (HR-TEM) micrograph of Co@C-SiO_2-*773* (scale bar 5 nm), (h) Co@C-SiO_2-*873* (scale bar 5 nm), and (i) Co@C-SiO_2-*973* (scale bar 5 nm).

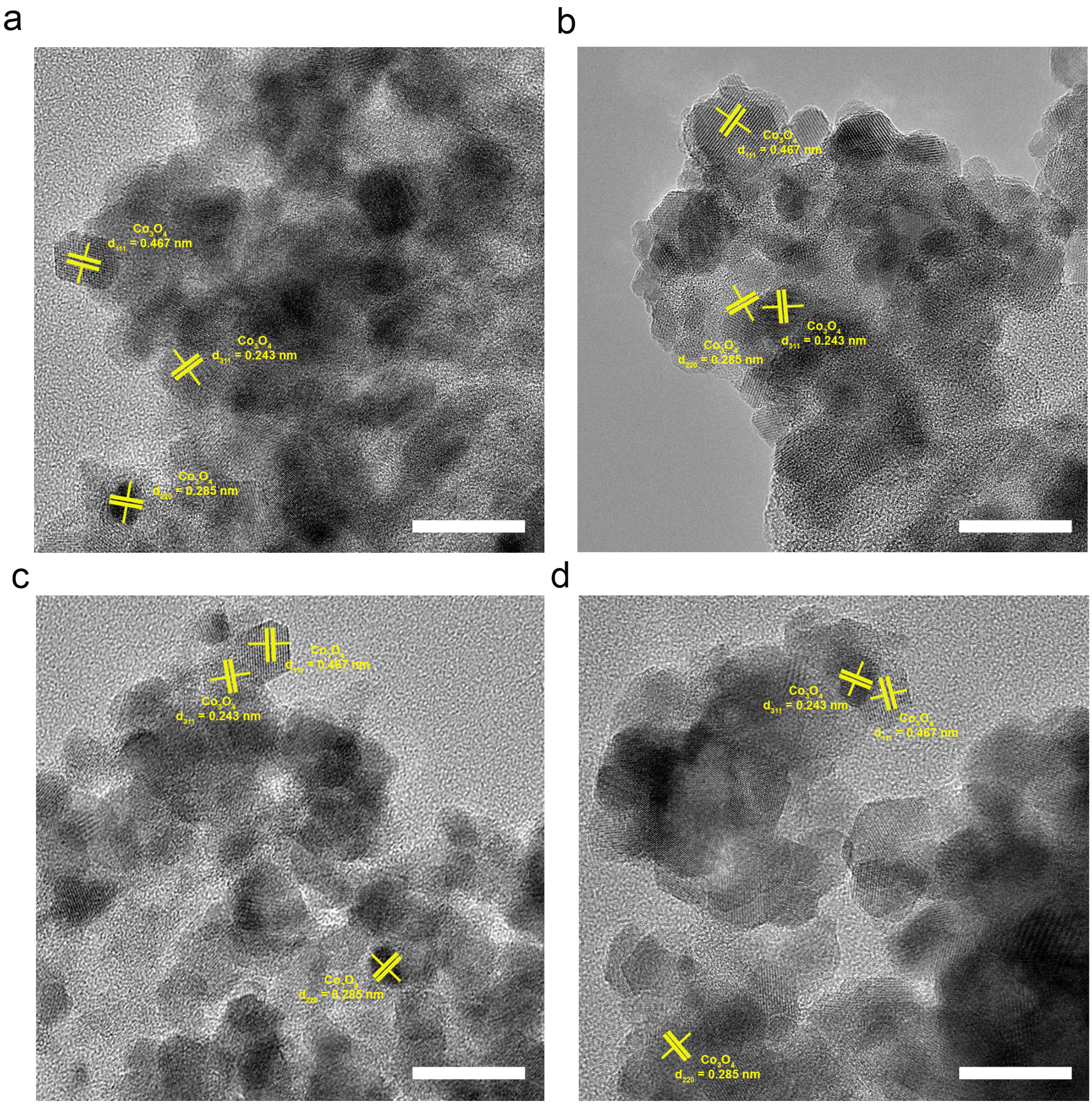

Supplementary Figure 6 High-resolution TEM (HR-TEM) micrographs of Co@SiO_2-*T* catalysts. (a) Co@SiO_2-*cal.,* (b) Co@SiO_2-*773*, and (c) Co@SiO_2-*873*. (d) Co@SiO_2-*973*. All scale bars 20 nm.

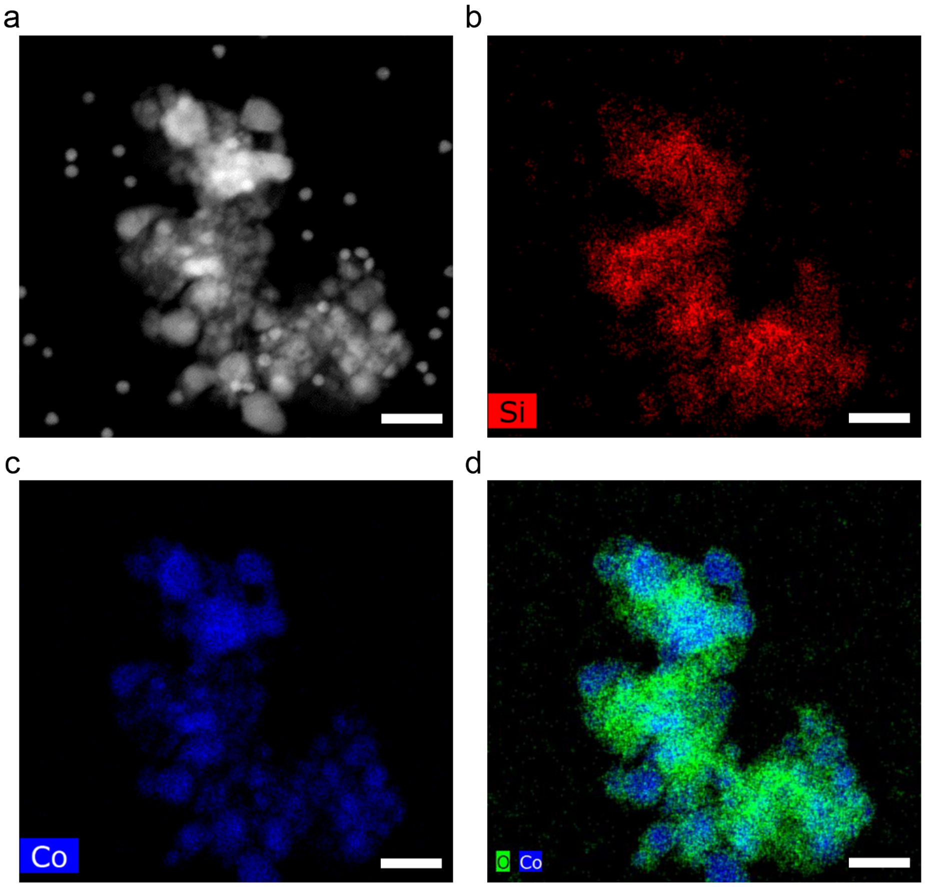

Supplementary Figure 7 TEM analysis of Co@SiO_2-*873* after reduction in 10 vol.% H_2/Ar (30 ml min^{-1}, STP) at 673 K for 10 h at a heating rate of 2 K min^{-1}, followed by passivation in 6 vol.% O_2/Ar (50 ml min^{-1}, STP) at 303 K for 2 h. (a) HAADF-STEM micrograph, (b) Elemental mapping of Si, (c) of Co, and (d) of Co (*blue*) and O (*green*) in the reduced Co@SiO_2-*873* sample. All scale bars 50 nm.

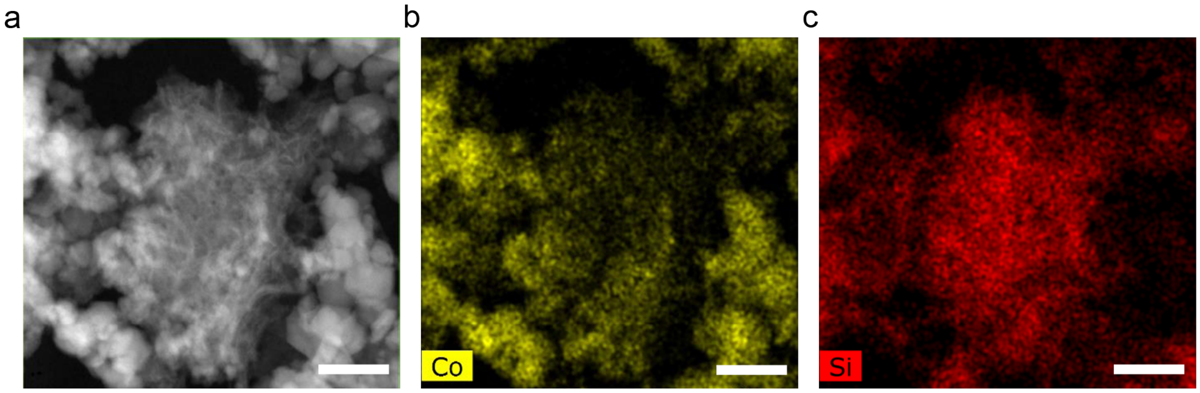

Supplementary Figure 8 TEM analysis of Co@SiO_2-*cal*. catalyst. (a) Dark field TEM micrograph, (b) Elemental mapping of Co, and (c) of Si. All scale bars 30 nm.

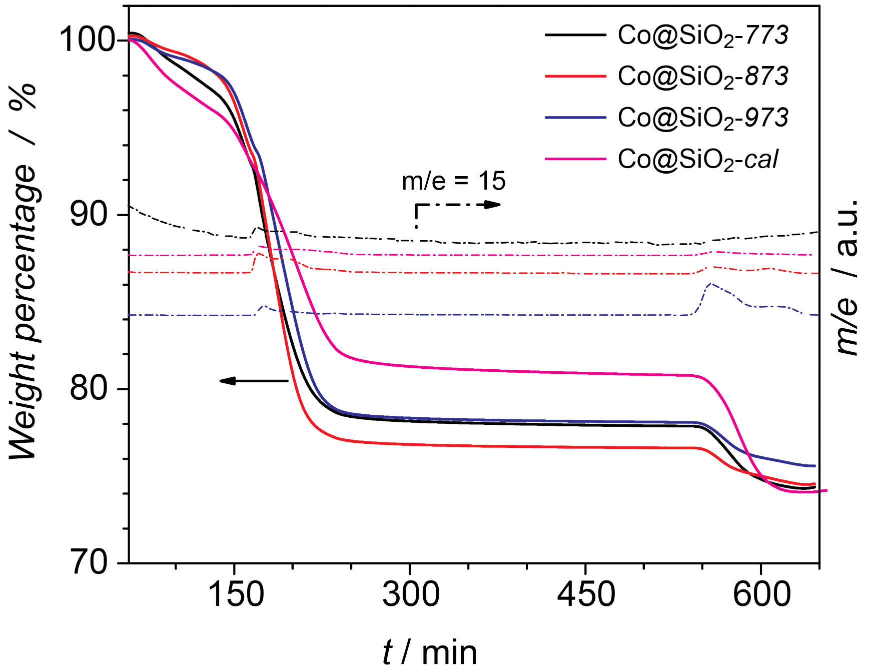

Supplementary Figure 9 Thermogravimetric (TG) analysis (solid lines) of Co@SiO_2 catalysts in a flow of 10 vol.% H_2/Ar combined with mass spectroscopic (MS) gas phase analysis for *m/e* = 15 (CH_3^+) corresponding to methane (dashed lines). Samples of ~10 mg Co@SiO_2 catalysts were used. The temperature was increased from room temperature to 673 K (5 K min^{-1}), held there for 5 h and then further increased to 1223 K (10 K min^{-1}).

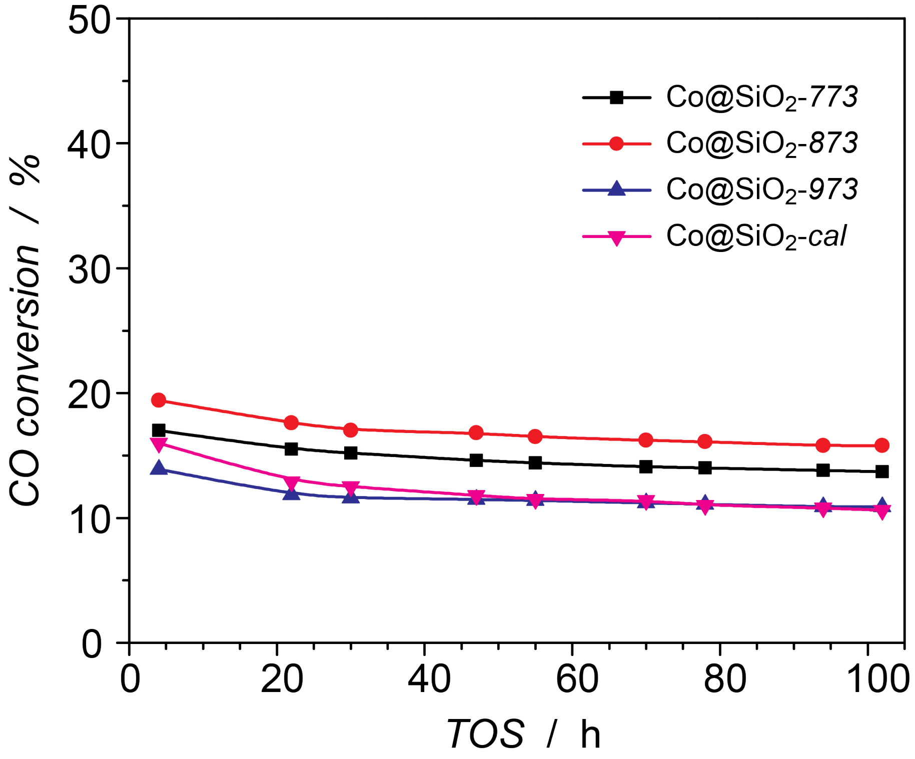

Supplementary Figure 10 CO conversion as a function of time-on-stream (*TOS*) in the FTS over Co@SiO_2-*T* and Co@SiO_2-*cal*. Reaction conditions: 483 K, 20 bar, H_2/CO = 1, 100 mg catalysts and syngas flow of 40 ml min^{-1}.

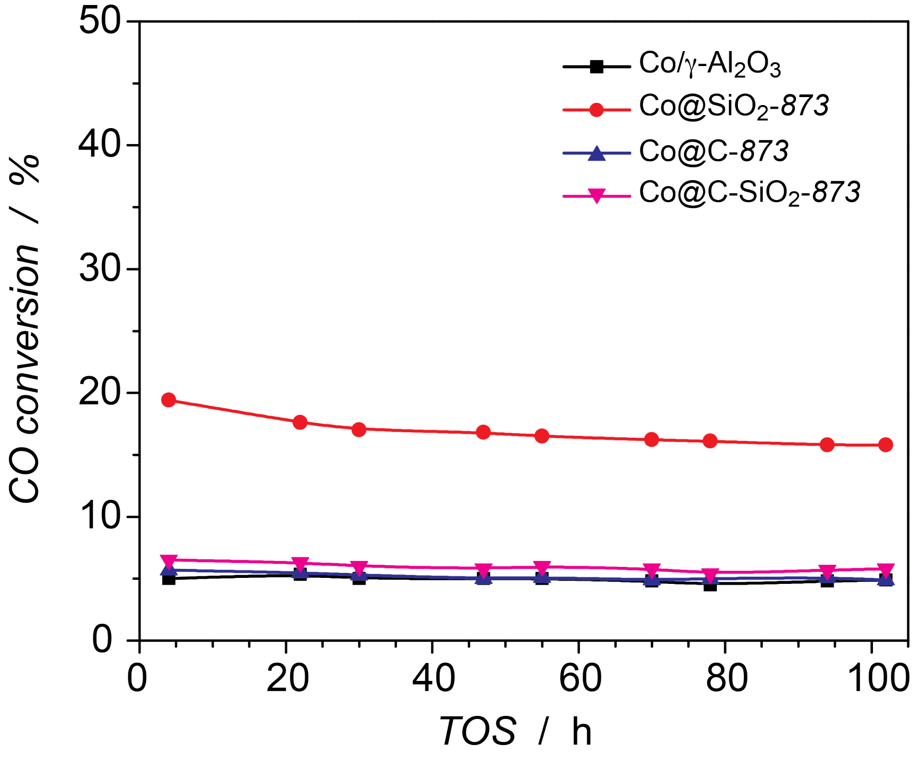

Supplementary Figure 11 CO conversion as a function of time-on-stream (*TOS*) in the FTS over Co/γ-Al_2O_3, Co@SiO_2-*873*, Co@C-*873*, and Co@C-SiO_2-*873* catalysts. Reaction conditions: 483 K, 20 bar, H_2/CO = 1, and syngas flow of 40 ml min^{-1}.

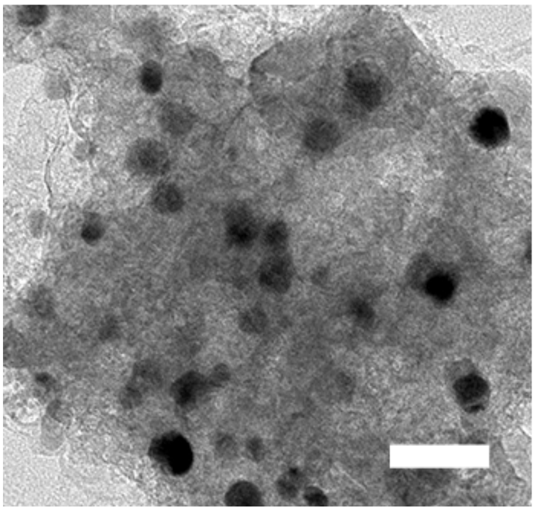

Supplementary Figure 12 TEM analysis of Co@C-*873(al)* catalyst (scale bar 50 nm).

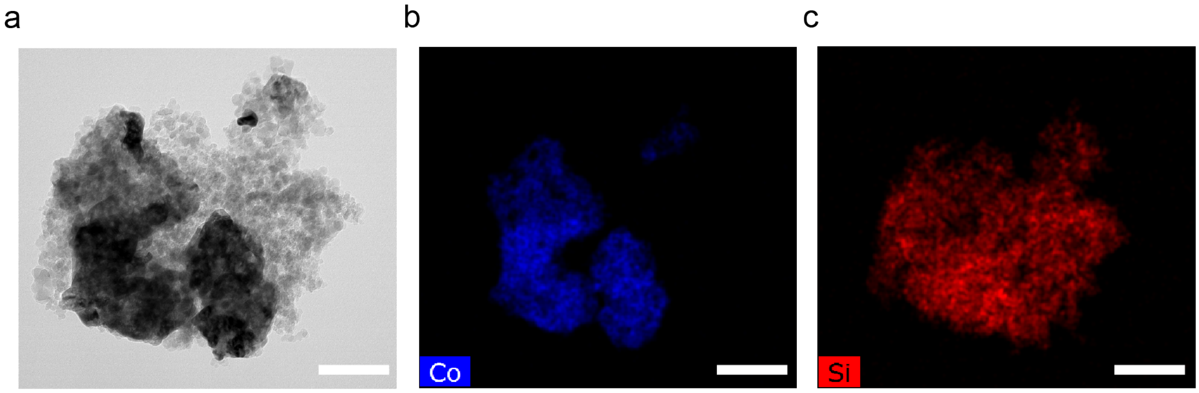

Supplementary Figure 13 TEM analysis of Co/SiO_2-*A-MI* catalyst. (a) Bright field TEM micrograph, (b) Elemental mapping of Co, and (c) of Si. All scale bars 100 nm.

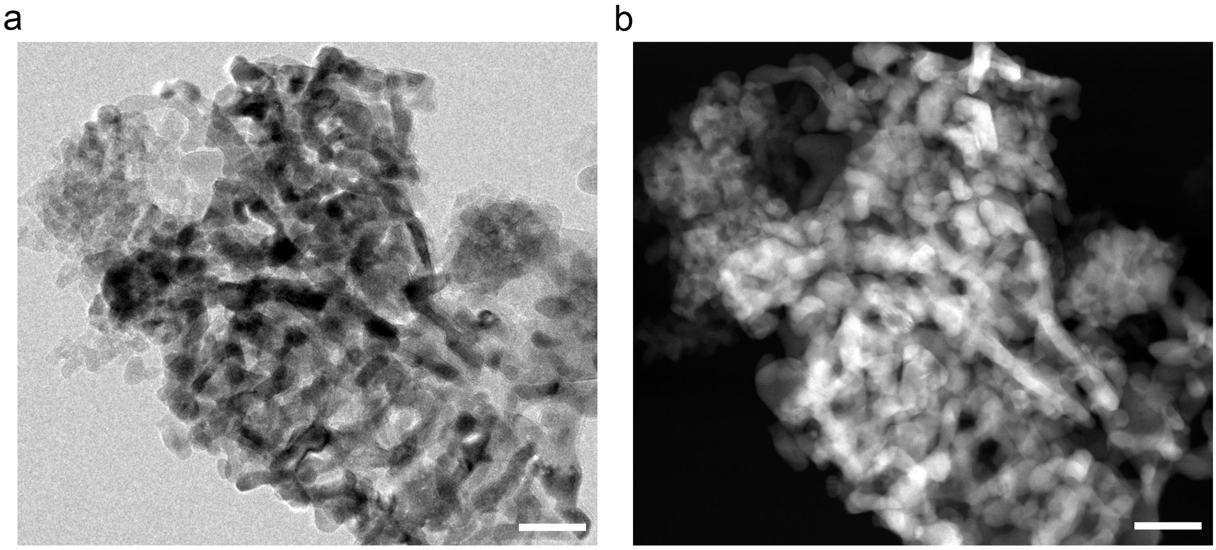

Supplementary Figure 14 Bright field (a) and dark field (b) TEM micrograph of Co/SiO_2-*F-TIMI* catalyst. All scale bars 50 nm.

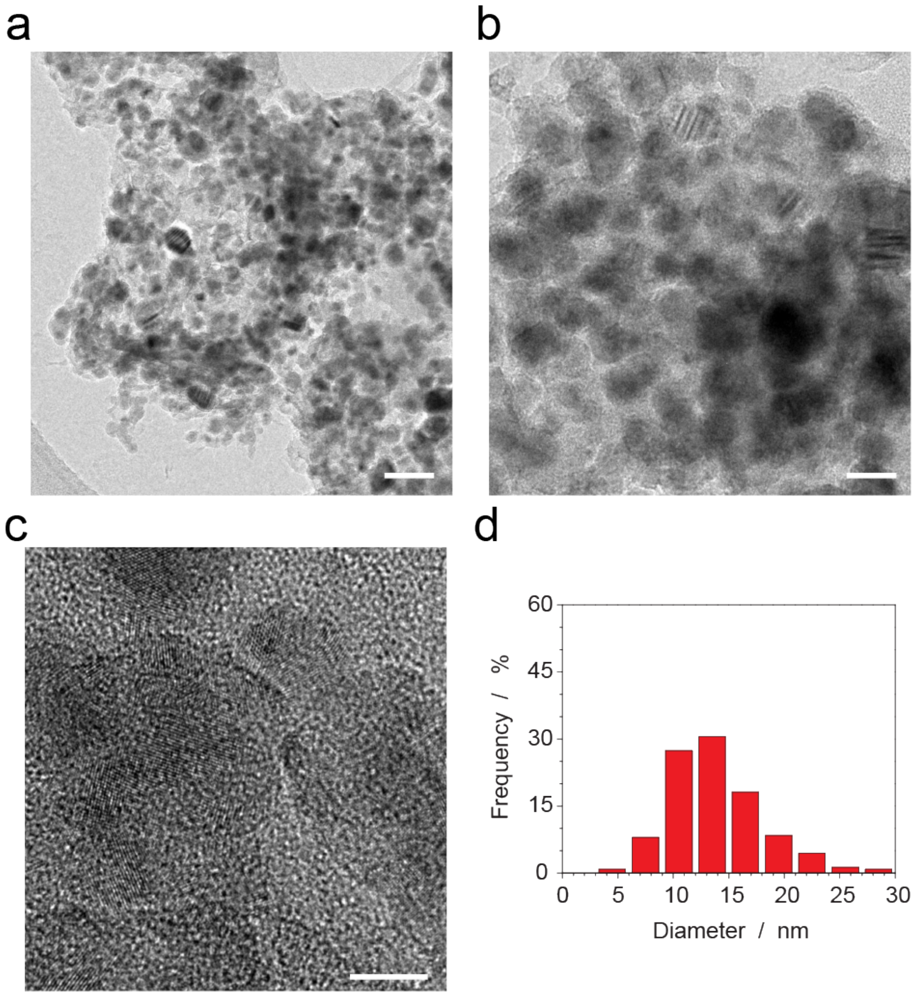

Supplementary Figure 15 Bright field (a,b) and high-resolution (c) TEM micrograph of Co@SiO_2-*873-spent* catalyst (scale bar: a: 50 nm; b: 20 nm; c: 5 nm). (d) Particle size

histograms obtained from TEM analysis for Co@SiO_2-*873-spent* catalyst, with an average particle size of 16 nm.
