# Supplementary material for: Manufacture of highly loaded silica-supported cobalt Fischer–Tropsch catalysts from a metal organic framework
Source: Nat Commun. 2017 Nov 22;8:1680. doi: 10.1038/s41467-017-01910-9 (PMC5698480; doi:10.1038/s41467-017-01910-9)
Supplement: Supplementary file 1 — Peer Review File [file 41467_2017_1910_MOESM1_ESM.pdf]

Reviewer #1 (Remarks to the Author):

This manuscript describes the synthesis of highly loaded and active silica supported cobalt Fischer-Tropsch catalysts using metal organic frameworks as sacrificial templates. The authors state that they present a stepwise methodology, which allows addressing the long-standing challenge in preparing highly loaded metal nanoparticle supported catalysts. It is really an overstatement, because similar Co-C-N based catalysts prepared by using ZIF-67 as sacrificial template and their catalysis application have already been reported a few years ago, where their properties such as metal loading, nanoparticle size, distribution and accessibility could be also well controlled by adjusting the synthetic route and pyrolysis temperature (e.g. Adv. Energy Mater. 2016, 6, 1501497; Adv. Mater. 2015, 27, 5010; J. Mater. Chem. A, 2014, 2, 14064; Chem. Commun., 2015, 51, 8292). The only innovation of this manuscript is to use SiO_2 as an additive template to prepare their silica-Co FTS catalysts, but there are already several articles reporting fairly similar or the same approach of using MOFs-SiO_2 composites as templates towards the synthesis of highly-dispersed SiO_2 supported catalysts to that reported herein (e.g. Angew. Chem. Int. Ed., 2016, 128, 1092). In addition, the catalytic properties of the Co@SiO_2 catalysts reported by the authors are by no means surpassing those of state-of-the-art Co-based FTS catalysts. There are also several much more reactive Fe-based FTS catalysts known as compared to the Co-SiO_2 catalysts reported here. Thus, either from the manufacturing side or from the property side, the method and materials reported here did ot lead to an improvement of existing technology. Therefore, this manuscript has insufficient novelty and technical quality to be published in Nat. Commun.

Reviewer #2 (Remarks to the Author):

Manuscript entitled " Stepwise synthesis of highly loaded and active Silica supported Cobalt Fischer-Tropsch catalysts using Metal Organic Frameworks as sacrificial templates" it has been synthesized with a number of different methods and in a variety of forms. This manuscript does not bring any new knowledge or data on materials property and therefore only contribution may be in novel preparation method, still this point is not elaborated properly (see Remark 1).Structure-activity relation is ambiguous; there are several statements not supported with data (for some see Remarks 2) and even some flaws (see Remark 3). For these reasons I recommend that the manuscript is rejected from being published in the Nature Communication.

1. This work described Co@SiO_2, Co@SiO_2-cal, Co/SiO_2-A-MI and Co/SiO_2-F-MI samples' various performance during FTO reaction and tried to explain the structure-activity relation through bulk characterization. There already are a lot of papers reported influence of the silica on the behaviors of the cobalt catalysts, excepted for the novel metal organic frameworks as sacrificial templates. Therefore, the novelty of the article is not enough.
2. The Table 2 show that the selectivity of C5 + nearly 90%, the selectivity for Co@SiO_2 is too good to be true. Can the authors give the full product distribution as supplementary material? What is the chain growth factor? Even worse, the authors did not discuss structure-activity relationships in detail. According to experience, Co@C-SiO_2 (Figure S3) at 973k is easy to form Co_2C, please carefully compare the PDF card.
3. The authors said the metal cobalt is the active phase in FTS of C5+, and many of the literature has shown that the carbon material and metal cobalt is moderate, easy to cobalt reduction, according to XRD results Co@C-SiO_2 (Figure S3) phase is cobalt, Why not directly use Co@C-SiO_2 as FTS catalyst is puzzling. But the author in turn the sample air was burned to remove carbon, get Co_3O_4@SiO_2, according to the H_2-TPR(Figure 3) results show that the reduction of cobalt is only 79%, Does this reduce the active site?

Minor points:
- Many experimental procedures are not described sufficiently precise. Which specific BET method was used for surface area calculation?

- No HR-TEM micrograph of the Co@SiO2 catalyst
- If the product distribution follows the ASF model, what is the chain growth probability?

Reviewer #3 (Remarks to the Author):

The authors have presented an approach t prepare high Co loaded FT catalysts. The manuscript is well written and merits publication.

It would be helpful if the authrs provide a comparison of their catalyst to a more traditionally prepared Co-alumina catalyst.

It would be helpful if the authors provide some discussion about their method to prepare high Co loaded catalyst and compare theirs to those of Velocysis and Johnson Matthy Catalysts workers.

Rebuttal

X. Sun *et al.*, Stepwise synthesis of highly loaded and active Silica supported Cobalt Fischer-Tropsch catalysts using Metal Organic Frameworks as sacrificial templates.

Bold-italics text: Comments by the reviewers

Regular text: Reply to the reviewers

Green text: Main text of the manuscript. Bold highlights the modifications and/or text added

Blue text: Supplementary Information, Bold highlights the modifications and/or text added

---

Reviewer #1 (Remarks to the Author):

This manuscript describes the synthesis of highly loaded and active silica supported cobalt Fischer-Tropsch catalysts using metal organic frameworks as sacrificial templates. The authors state that they present a stepwise methodology, which allows addressing the long-standing challenge in preparing highly loaded metal nanoparticle supported catalysts. It is really an overstatement, because similar Co-C-N based catalysts prepared by using ZIF-67 as sacrificial template and their catalysis application have already been reported a few years ago, where their properties such as metal loading, nanoparticle size, distribution and accessibility could be also well controlled by adjusting the synthetic route and pyrolysis temperature (e.g. Adv. Energy Mater. 2016, 6, 1501497; Adv. Mater. 2015, 27, 5010; J. Mater. Chem. A, 2014, 2, 14064; Chem. Commun., 2015, 51, 8292).

We agree that highly loaded metal@C catalysts can be prepared by pyrolyzing MOFs under inert atmosphere with controllable nanoparticle size and distribution. However, when it comes to transition metals such as cobalt, nickel and copper etc., the accessibility of these nanoparticles should be carefully taken into consideration. In case of cobalt, it is well known that during the pyrolysis of Co-MOFs, the cobalt cations in the framework will be reduced and form metallic cobalt nanoparticles. These metallic cobalt nanoparticles further catalyse the formation of graphitic layers under high temperature pyrolysis and subsequent cooling down. These graphitic layers encapsulate the formed cobalt nanoparticles and render them inaccessible. In order to further demonstrate this point, we have added catalytic results of directly pyrolysed ZIF-67 and ZIF-67@SiO_2, namely Co@C-*873* and Co@C-SiO_2-*873*. These results have been added to the supplementary information file (SI), and are summarized in the new Figure S10 and Table S2. The poor accessibility of cobalt nanoparticles in the directly pyrolyzed sample Co@C-*873* is also proven by acid leaching experiment, as present in Table S3 and Figure S12 in the SI file.

Accompanying the figure and tables, the following text has been added to the supporting information (SI).

<< CO conversion as a function of time-on-stream (TOS) in the FTS over Co@C-*873*, and Co@C-SiO_2-*873* catalysts is shown in Figure S10. Reaction conditions: 483 K, 20 bar, H_2/CO = 1, and syngas flow of 40 ml min^{-1}. All these catalysts show much lower CO conversion in FTS than the Co@SiO_2-*873* catalyst.>>(Page 9)

<<Catalytic performance of Co@C-*873*, and Co@C-SiO_2-*873* catalysts after 102 h TOS is shown in Table S2. Co/γ-Al_2O_3 was prepared using incipient wetness impregnation method with aqueous cobalt nitrate solution, followed by drying at 373 K under vacuum overnight and calcination under air flow (150 ml min^{-1}) for 2 h at a ramp rate of 1 K min^{-1}. Carbon conversion (X_{CO}, %), activity per gram of Co (*CTY*), hydrocarbon selectivity (*S*, %). FTS experiments were carried out at 483 K, 20 bar, and H_2/CO = 1, and syngas flow of 40 ml min^{-1}. All these directly pyrolyzed catalysts show much lower cobalt-time-yield (*CTY*) and C5+ selectivity but much higher undesired CH_4 selectivity.>>(Page 10)

<< Cobalt loading in Co@C-*873* and Co@C-*873(al)* catalysts is shown in Table S3. Co@C-*873(al)* was obtained by immersing 0.5 g Co@C-*873* in 500 ml of 0.5 M hydrochloric acid solution for 4 days at 303 K to dissolve the exposed (accessible) cobalt nanoparticles, followed by washing with deionized water and drying at 323 K under vacuum. Almost 70 wt.% cobalt is completely encapsulated by graphitic shells which render them inaccessible.>>(Page 10)

The following text has been added to the main article:

<<In addition, although pyrolysis of Co based MOFs under an inert atmosphere has recently been demonstrated as a promising route to prepare highly loaded Co@C hybrids with controllable cobalt particle size and distribution, these directly pyrolyzed samples such as Co@C-*873* and Co@C-SiO_2-*873* synthesized in this work show a poor activity and low C5+ selectivity along with an unacceptable CH_4 selectivity in the FTS process under the same conditions as Co@SiO_2 catalysts (Supplementary Fig. 11 and Supplementary Table 2). The inferior performance of these pyrolyzed samples can be ascribed to the inaccessibility of most cobalt nanoparticles, which are completely encapsulated by graphitic shells (Supplementary Fig. 12 and Supplementary Table 3). >> (Page 9)

The only innovation of this manuscript is to use SiO_2 as an additive template to prepare their silica-Co FTS catalysts, but there are already several articles reporting fairly similar or the same approach of using MOFs-SiO_2 composites as templates towards the synthesis of highly-dispersed SiO_2 supported catalysts to that reported herein (e.g. Angew. Chem. Int. Ed.,2016,128,1092).

We disagree with the view of the referee. First of all, the approach here presented solves the issue highlighted above. Furthermore, in contrast to previous publications, SiO_2 here acts as a catalyst support, not as an additive. Regarding the reference mentioned by the referee, we would like to highlight here several differences: (1) The cobalt loading in this reference is only 19 wt.%, a moderate cobalt loading and much lower than ours (~50 wt.%); (2) The cobalt nanoparticle size in this reference is smaller than 2 nm. On one hand, such small cobalt nanoparticles will have a very strong interaction with the SiO_2 support and can be hardly reduced. On the other hand, considering that FTS is a structure sensitive reaction, cobalt nanoparticles with sizes below ~6 nm normally exhibit poor activity and stability (i.e. easy reoxidation and aggregation etc.), and a high, undesired CH_4 selectivity. Thus, the reported Co@SiO_2 catalyst in the reference can hardly be applied in FTS. In contrast, our hydrolysis-pyrolysis-calcination strategy can well control cobalt particle size above 6 nm, together with a good distribution (vs melt infiltration method) and high cobalt reducibility (vs deposition-precipitation method), even at such a high cobalt loading (~50 wt.%). This comparison further highlights the importance and novelty of our work.

In addition, the catalytic properties of the Co@SiO_2 catalysts reported by the authors are by no means surpassing those of state-of-the-art Co-based FTS catalysts. There are also several much more reactive Fe-based FTS catalysts known as compared to the Co-SiO_2 catalysts reported here. Thus, either from the manufacturing side or from the property side, the method and materials reported here did not lead to an improvement of existing technology. Therefore, this manuscript has insufficient novelty and technical quality to be published in Nat.Commun.

We strongly disagree with the reviewer and, in the next few lines, we will demonstrate that these statements are plainly wrong. First of all, Fe catalysts show almost no activity under low temperature FTS conditions, that is the reason why Co is the state-of-the-art for the direct formation of waxes. Assuming that the reviewer is familiar with the field of Fischer Tropsch synthesis, we are very curious and would like to challenge him/her to come up with references that provide evidence for a statement such as "*much more reactive Fe-based FTS catalysts known as compared to the Co-SiO_2 catalysts reported here*".

In addition to the comment on much more reactive Fe-based catalysts, the referee states that our results by no means surpass state of the art Co based catalysts. Also here appropriate citations of literature that reports similar highly loaded Co systems displaying a higher activity than the catalysts here reported should be given by the referee. From our side, in order to demonstrate the high activity, stability and C5+ selectivity of the Co@SiO_2 in this work,

we compared our $Co@SiO_2$-*873* catalyst with Co/SiO_2 catalysts prepared following conventional (state of the art) methods, including incipient wetness impregnation and melt infiltration methods. We have already mentioned in the introduction section why we use these two preparation methods: due to the formation of a large amount of irreducible cobalt silicate species in the deposition-precipitation method. One can clearly see from Table 3 in our manuscript, that the cobalt-time-yield of the highly loaded $Co@SiO_2$-*873* (4.2 $*10^{-5}$ $mol_{CO}g^{-1}_{Co}s^{-1}$) prepared by using this new strategy exceeds all the other reference $Co@SiO_2$ catalysts, irrespective of the Co/SiO_2 with a normal cobalt loading of 16.5 wt.% (3.1 $*10^{-5}$ $mol_{CO}g^{-1}_{Co}s^{-1}$) or with much higher cobalt loading of 42 wt.% (3.0 $*10^{-5}$ $mol_{CO}g^{-1}_{Co}s^{-1}$). This further demonstrates that the highly loaded supported cobalt catalyst prepared with this new strategy shows an excellent FTS performance. Please be aware of the conditions that we used in this work: 210 ^{o}C, $H_2/CO=1$, and, to the best of our knowledge, no better catalysts has been reported in the literature able to show such performance under these mild conditions.

Reviewer #2 (Remarks to the Author):

Manuscript entitled "Stepwise synthesis of highly loaded and active Silica supported Cobalt Fischer-Tropsch catalysts using Metal Organic Frameworks as sacrificial templates" it has been synthesized with a number of different methods and in a variety of forms. This manuscript does not bring any new knowledge or data on materials property and therefore only contribution may be in novel preparation method, still this point is not elaborated properly (see Remark 1). Structure-activity relation is ambiguous; there are several statements not supported with data (for some see Remarks 2) and even some flaws (see Remark 3). For these reasons I recommend that the manuscript is rejected from being published in the Nature Communication.

1. This work described $Co@SiO_2$, $Co@SiO_2$-cal, Co/SiO_2-A-MI and Co/SiO_2-F-MI samples' various performance during FTO reaction and tried to explain the structure-activity relation through bulk characterization. There already are a lot of papers reported influence of the silica on the behaviors of the cobalt catalysts, excepted for the novel metal organic frameworks as sacrificial templates. Therefore, the novelty of the article is not enough.

We would like to draw the attention of the Editor to the fact that Reviewer 2 did not refer correctly to the content of our study:

- First of all, we report the preparation of Fischer-Tropsch Synthesis (FTS) catalysts based on Co that are applied at 210 ^{o}C and H_2/CO = 1 with the primary objective of realizing high chain growth probabilities and therefore form long chain hydrocarbons. The reviewer mentions that we report performance in FTO, Fischer-Tropsch to

Olefins. This process, carried out at circa 340 ^{o}C over Fe-based catalysts targets maximizing production of short chain olefins.

- Indeed, SiO_2 is one of the archetypical supports used in Co based catalysts for this application, however, as stated in our introduction, traditional synthesis methods usually lead to a limiting Co-loading of circa 20 wt.%, an issue that we solve with the approach here presented.

2. The Table 2 show that the selectivity of C5 + nearly 90%, the selectivity for Co@SiO$_2$ is too good to be true. Can the authors give the full product distribution as supplementary material? What is the chain growth factor? Even worse, the authors did not discuss structure-activity relationships in detail. According to experience, Co@C-SiO$_2$ (Figure S3) at 973k is easy to form Co$_2$C, please carefully compare the PDF card.

We again feel uncomfortable with this comment. By stating that our results are "too good to be true", the reviewer sheds doubts on the correctness of the results, while at the same time highlighting his/her unfamiliarity about some recent related literature. The presented selectivities are at the level of some of the best FTS Co-catalysts previously published (see for instance: **ACS Catal., 2016, 6, 1616-1623; J. Catal., 2015, 328, 130-138; or J. Am. Chem. Soc., 2014, 136, 7333-7340.**), indicating the significance of this result having been obtained at these high Co-loadings.

In order to further demonstrate our claims and to erase any doubt about this research, a thorough analysis of the collected products has been performed by collaborators at Shell Global Solutions. The product distribution and chain growth factor for the best Co@SiO$_2$-*873* catalyst was added in the main article, as shown in Figure 5. The graph clearly shows that hydrocarbon products in the range of C1-C100 are formed. From this graph, a chain growth probability (α) as high as 0.94 has been determined.

The following text has been added to the main article:

<< Interestingly, the Co@SiO$_2$-*873* exhibits a chain growth probability (α) as high as 0.94, further confirming the high C5+ selectivity in this work, as shown in Figure 5.>> (Page 9)

In addition, the structure-activity relationships of Co@SiO$_2$ catalysts are discussed in detail in the main article:

<< The FTS process occurs on the surface of metallic cobalt nanoparticles with an optimal particle size around 10 nm. On one hand, small cobalt nanoparticles normally possess a large fraction of low-coordinated surface sites (i.e. corner, kink, edge etc.), which to a large extent hamper CO dissociation and/or CH$_x$ hydrogenation. Hence, we attribute the superior activity of Co@SiO$_2$-*873* to the high Co reducibility and the optimal Co particle size (Table 1). On the other hand, small cobalt nanoparticles have only few step sites, known for C-C formation towards long chain hydrocarbons,

therefore resulting in a high methane selectivity. Thus, the larger Co-particle size in the Co@SiO_2-*873* and Co@SiO_2-*973* samples when compared to Co@SiO_2-*773* results in a lower CH_4 and a higher C5+ selectivity for these catalysts (Table 2), in excellent agreement with literature. >> (Page 9)

From XRD analysis, we can clearly see that there are only graphite and metallic cobalt phases in the Co@C-SiO_2-*973* sample. In general, cobalt carbide is not stable at high temperature and can easily decompose and transform to graphite covering the surface of metallic cobalt nanoparticles (see references: **Nature, 1996, 381, 772-774; J. Phys. Soc. Jpn. 16, 1961, 1213-1219**). These graphitic shells prevent the oxidation of metallic cobalt cores under air conditions; therefore only metallic cobalt phase is detected in XRD analysis.

3. The authors said the metal cobalt is the active phase in FTS of C5+, and many of the literature has shown that the carbon material and metal cobalt is moderate, easy to cobalt reduction, according to XRD results Co@C-SiO_2 (Figure S3) phase is cobalt, Why not directly use Co@C-SiO_2 as FTS catalyst is puzzling. But the author in turn the sample air was burned to remove carbon, get Co_3O_4@SiO_2, according to the H_2-TPR(Figure 3) results show that the reduction of cobalt is only 79%, Does this reduce the active site?

We added the experimental data of the Co@C-SiO_2-*873* catalyst treated under the same FTS conditions in Figure S11 and Table S2 in the supporting information. As we can see, before burning off the carbon materials, this sample exhibits a very poor activity (cobalt-time-yield) and low C5+ selectivity but a high, undesired CH_4 selectivity. This again demonstrates that the sample after pyrolysis is not suitable for FTS, and further highlights the importance of the calcination step afterwards.

In addition, compared to the 'direct-calcination' sample (Co@SiO_2-*cal.*) the intermediate pyrolysis step between hydrolysis and calcination is essential to improve the cobalt reduction degree and FT activity. This new preparation strategy increases the cobalt reduction degree to ~80%, a similar value as the impregnation method (but only with maximum cobalt loading of 20 wt.%) and much higher than the deposition-precipitation method (high cobalt loading but with substantial cobalt silicate), but for a ~50 wt.% cobalt content. All in all, the multi-step synthesis strategy solves the long standing problem about how to prepare highly loaded supported metal catalysts with high reducibility, good particle size distribution and accessibility, and good catalytic performance.

Minor points:

- ***Many experimental procedures are not described sufficiently precise. Which specific BET method was used for surface area calculation?***

  More N_2 adsorption information has been added into the main article:
  <<N_2 adsorption-desorption isotherms were obtained using a Micromeritics Tristar 3020 at 77 K, and samples were outgassed under vacuum at 423 K overnight prior to the analysis. The BET area was determined as outlined in ref. 32 in the main article. The mesopore surface area was obtained from the *t*-plot applied to the N_2 isotherm. >> (Page 11)

- ***No HR-TEM micrograph of the Co@SiO2 catalyst***

HR-TEM micrographs have been added to Figure S6 in the supporting information, and the following text has been added to the supporting information (SI).

<<High-resolution TEM (HR-TEM) micrographs of Co@SiO_2-*T* catalysts. (a) Co@SiO_2-*cal.*, (b) Co@SiO_2-*773*, and (c) Co@SiO_2-*873*. (d) Co@SiO_2-*973*. All scale bars 20 nm.>>(Page 6)

- ***If the product distribution follows the ASF model, what is the chain growth probability?***

The chain growth probability (α) is 0.94, calculated in the range of C15-C100, as shown in Figure 5.

Reviewer #3 (Remarks to the Author):

The authors have presented an approach to prepare high Co loaded FT catalysts. The manuscript is well written and merits publication.

It would be helpful if the authors provide a comparison of their catalyst to a more traditionally prepared Co-alumina catalyst.

It would be helpful if the authors provide some discussion about their method to prepare high Co loaded catalyst and compare theirs to those of Velocysis and Johnson Matthy Catalysts workers.

We thank Reviewer 3 for very favorable comments about our work. And we have already added the FTS data for a traditional Co/γ-Al_2O_3 catalyst prepared by using incipient wetness impregnation method with cobalt loading of 17 wt.% in the supporting information (Figure

S10 and Table S2). It is clearly seen that this Co/γ-Al_2O_3 catalyst shows a much poorer FTS performance than the reported Co@SiO_2-*873* catalyst in this work.

Reviewer #1 (Remarks to the Author):

The manuscript should be retruned to the authors so they can make a much improved response to the reviewer's comments.

For example, in response to Reviewer 1 comments the authors state that "Fe catalysts show almost no activity under low temperature FTS cibdutuibs..." This statement is absolutely not true. Sasol workers clearly show in graphical form where Co catalysts are superior and where Fe catalysts are superior.

Reviewer #2 (Remarks to the Author):

The manuscript by Gascon et al. presents the use of MOF as sacrificial templates for the preparation of cobalt based catalysts for Fischer – Tropsch synthesis. The authors report on the long standing challenge of catalyst preparation at high loadings of the active metal, a topic especially important for unlocking small scale applications of FTS technology. In addition, the manuscript paves the way for new approaches in breaking the interdependence of crucial structural parameters influencing FTS performance. The manuscript is well constructed and written having the potential to be published at Nature communications. However, clearly not all claims are supported by the presented data. Therefore I would recommend publication only after major revision. The major issues leading to this suggestion are summarized in the following points:
Authors claim better activity, selectivity to higher hydrocarbons and stability for the catalysts prepared by the MOF templating method.

1.The claim of excellent stability cannot stand with only the presented data. All tested catalysts appear to have very similar stability (Figure 10, supporting) with the exception of Co@SiO2-cal. catalyst that shows some deactivation. Therefore, it cannot be concluded that the presented preparation procedure outperforms in terms of stability catalytic materials prepared by conventional methods. In addition, and more importantly the tests have been performed at moderate to low carbon monoxide conversions (<20%) and consequently low partial pressures of steam. In Co based FTS it is well accepted that the initial deactivation rate is related to pH2O, consequently tests at more realistic conversions (>50%) would allow a clear statement on stability.

2.The effect of water in FTS is paramount. Apart from catalyst stability, the H2O formed affects both activity and selectivity. Conversion level has a positive influence into the C5+ selectivity therefore comparison of small differences (see Tables 2 and 3) is valid only for data obtained at similar conversion levels. In addition, a low H2 to CO ratio is used (H2/CO=1) that is far from the stoichiometric (2.1). This renders comparison with literature values unfair since low ratios are boosting C5+ up to 15%. Finally, the carbon removed as CO2 is not considered in the calculations increasing the error.

3.The comparison with other preparation techniques (IWI and MI) is unfair since the Co particle size is not evaluated, when these conventional techniques are used. Furthermore, the widely used and easily applied 2-step impregnation is missing from the conventional techniques. Why the authors choose to leave it outside comparison?

4.Finally the reasoning behind the enhanced performance (1.5 times higher CTY and a=0.94) are not discussed in terms of catalysts properties. Could they be explained simply by the particle size effect? Authors are not validating that the measured particle size with TEM or H2 chemisorption will remain the same after 20 bars of H2. Did the authors performed similar characterization on the spent catalyst?
For the above reasons I believe that the study needs significant rewriting and/or additional experimental work in order to convince on the clear benefit of the suggested preparation route.

Minor comments of consideration

•Figure 1 miss the H_2 reduction step

•Since the study refers to the structure-sensitivity of FTS a reference to the first paper discovering the phenomenon Journal of Catalysis 200, (2001) 106 in addition to refs 13 and 14 has to be included.

•Similarly the addition of the first paper describing the formation of needle-like structures of Cobalt and silica on FT catalyst by Exxon Journal of Catalysis 217 (2003) 127.

•Authors use XRD to claim that carbon is preventing oxidation by air. XRD probes long range order and oxidized Co nuclei of few layers would not be detected. This statement could only be made if in situ XRD would be used. It is surprising that the catalysts used directly after pyrolysis have some activity, since they are fully covered with carbon.

Rebuttal

X. Sun *et al.*, Stepwise synthesis of highly loaded and active Silica supported Cobalt Fischer-Tropsch catalysts using Metal Organic Frameworks as sacrificial templates.

Bold-italics text: Comments by the reviewers

Regular text: Reply to the reviewers

Green text: Main text of the manuscript. Bold highlights the modifications and/or text added

Blue text: Supplementary Information, Bold highlights the modifications and/or text added

---

Reviewer #1 (Remarks to the Author):

This manuscript should be returned to the authors so they can make a much improved response to the reviewer's comments

For example, in response to Reviewer 1 comments the authors state that 'Fe catalysts show almost no activity under low temperature FTS conditions…' This statement is absolutely not true. Sasol workers clearly show in graphical form where Co catalysts are superior and where Fe catalysts are superior.

We thank Reviewer #1 for his time to read once more our work. We agree with the reviewer that the statement should be modified. We have also modified the comparison between cobalt and iron catalysts in low-temperature Fischer-Tropsch synthesis, as presented below:

- Indeed, Co and Fe catalysts have both been applied in low-temperature Fischer-Tropsch processes, normally in the range between 200-250 ^{o}C. The reaction rate over cobalt catalysts however, is much higher than that over iron catalysts, indicating that the intrinsic activity of cobalt on a per gram catalyst basis is much higher than that of iron.[1, 2, 3]
- When iron catalysts are used under low-temperature FT conditions, reoxidization of the active phase and sintering of the metal crystallites caused by water are severe.[3] In this context, the higher resistance towards reoxidation for Co based catalysts as compared to Fe based catalysts allows for longer runs (for several years).[4] Furthermore, reoxidation of iron is also accompanied by a significant increase in CO_2 production, which is generally not desired.[3]

Reviewer #2 (Remarks to the Author):

The manuscript by Gascon et al. presents the use of MOF as sacrificial templates for the preparation of cobalt based catalysts for Fischer – Tropsch synthesis. The authors report on the long standing challenge of catalyst preparation at high loadings of the active metal, a topic especially important for unlocking small scale applications of FTS technology. In addition, the manuscript paves the way for new approaches in breaking the interdependence of crucial structural parameters influencing FTS performance. The manuscript is well constructed and written having the potential to be published at Nature communications. However, clearly not all claims are supported by the presented data. Therefore I would recommend publication only after major revision. The major issues leading to this suggestion are summarized in the following points: Authors claim better activity, selectivity to higher hydrocarbons and stability for the catalysts prepared by the MOF templating method.

We thank Reviewer #2 for the positive comments on our work.

1: The claim of excellent stability cannot stand with only the presented data. All tested catalysts appear to have very similar stability (Figure 10, supporting) with the exception of Co@SiO2-cal. catalyst that shows some deactivation. Therefore, it cannot be concluded that the presented preparation procedure outperforms in terms of stability catalytic materials prepared by conventional methods. In addition, and more importantly the tests have been performed at moderate to low carbon monoxide conversions (<20%) and consequently low partial pressures of steam. In Co based FTS it is well accepted that the initial deactivation rate is related to pH2O, consequently tests at more realistic conversions (>50%) would allow a clear statement on stability.

In order to show the stability of our Co@SiO_2-*873* catalyst at a higher CO conversion level, we operated the reaction with H_2/CO ratio of 2, and targeted an initial CO conversion above 75%. In this sense, a P_{H2O}/P_{H2} ratio of ~1.5 can be expected during the FT process, according to a recent thermodynamic calculation.[5] At these harsh conditions, cobalt nanoparticles smaller than 4 nm have been often reported to oxidize. These new data have been added in Figure 5b and Table 3 in the main article. As it can be observed, the behavior of the Co@SiO_2-*873* catalysts under such harsh reaction conditions does not differ from data at lower conversion levels.

2. The effect of water in FTS is paramount. Apart from catalyst stability, the H2O formed affects both activity and selectivity. Conversion level has a positive influence into the C5+ selectivity therefore comparison of small differences (see Tables 2 and 3) is valid only for data obtained at similar conversion levels. In addition, a low H2 to CO ratio is used (H2/CO=1) that is far from the stoichiometric (2.1). This renders comparison with literature values unfair since low ratios are boosting C5+ up to 15%. Finally, the carbon removed as CO2 is not considered in the calculations increasing the error.

We agree with Reviewer #2 that conversion level has a positive influence on C5+ selectivity. Indeed, it is well known that CH_4 selectivity is higher and C5+ selectivity lower at low conversion levels because of the higher H_2 partial pressure at these conditions.[6, 7, 8] On the other hand, cobalt particle size has also a significant effect on product selectivity. Cobalt nanoparticles with particle size above 8-10 nm normally exhibit a lower CH_4 and higher C5+ selectivity.[9] In spite of a lower CO conversion, Co@SiO_2-*973* still shows lower CH_4 and higher C5+ selectivity than Co@SiO_2-*773*, attributed to the larger particle size of cobalt nanoparticles in Co@SiO_2-*973*. Similarly, TEM analysis in Figure 2 confirms that the Co@SiO_2-*773* sample has much less cobalt nanoparticles with particle size above 8-10 nm than Co@SiO_2-*873*.

As suggested by Reviewer #2, in order to show a better comparison of our Co@SiO_2-873 with conventional Co/SiO_2 catalysts, we have performed a series of catalytic tests using a H_2/CO ratio of 2, as shown in Figure 5b and Table 3 in the main article. The Co@SiO_2-*873* catalyst displays a cobalt-time-yield of $7.8*10^{-5}$ $mol_{CO}g^{-1}{}_{Co}s^{-1}$ and C5+ selectivity of 84.7% after 100 h time-on-stream, with CO_2 selectivity lower than 1%, supporting the low water-gas-shift activity of cobalt catalyst. After 100 h time-on-stream, we changed the syngas flow and stabilize the reaction until 118 h *TOS* to obtain a CO conversion of 26%, as shown in entry 6 and 8 in Table 3. As it can be observed, under these conditions, the Co@SiO_2-*873* catalyst shows a C5+ selectivity of 82.8%, which is similar to that for the 32 wt.%Co/SiO_2 catalyst (83.0%) prepared by using a two-step incipient wetness impregnation method.

Thus, based on all the additional experiments, we feel confident to draw the conclusion that the Co@SiO_2-*873* catalyst prepared using MOF-mediated synthesis approach exhibits higher activity than the Co/SiO_2 catalyst prepared by conventional methods and a comparable C5+ selectivity to the Co/SiO_2 catalyst prepared by *IWI*.

The following text has been added to the main article:

<< Also two benchmark Co/SiO_2 catalysts with cobalt loading of 16 wt.% and 32 wt.%, respectively were prepared by means of incipient wetness impregnation (IWI). The 32 wt.%Co/SiO_2-*F-TIWI* and 40 wt.%Co/SiO_2-*A-MI* catalysts consist mostly of large aggregates (Supplementary Fig. 13a-c, and Supplementary Fig. 14a,b) as a result of the lower versatility of the MI and IWI methods for high cobalt loadings. The comparison between the FTS performance of these catalysts and Co@SiO_2-*873* is shown in Figure 5a,b and Table 3. Under the studied conditions, the Co@SiO_2-*873* displays a *CTY* at least 1.5 times (H_2/CO = 1) (entry 1-4, Table 3) and/or 2.2 times (H_2/CO = 2) (entry 5 and 7, Table 3) higher than the other samples (in spite of the higher Co loading) and a comparable C5+ selectivity (~ 83%) to its Co/SiO_2-*F-TIWI* counterpart at a similar CO conversion level (~ 26%). Interestingly, TEM images of the Co@SiO_2-*873* catalyst after 201 h *TOS* show a very good dispersion of cobalt nanoparticles on the SiO_2 support and hardly any aggregate (Supplementary Fig. 15a-d), in good agreement with the observed very mild catalyst deactivation with time on stream.>> (Page 10)

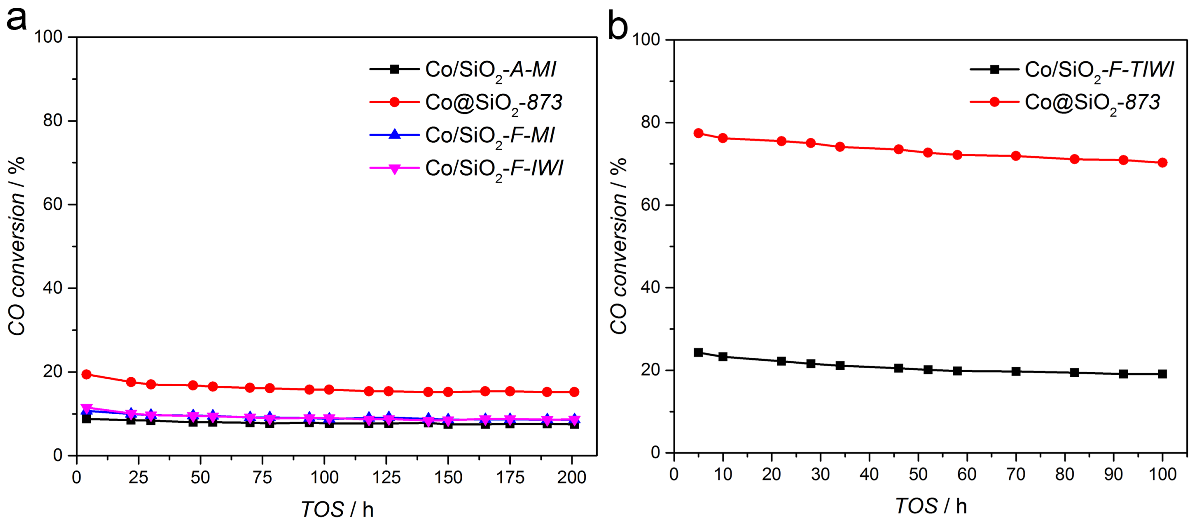

Figure 5 | Catalytic performance. (a) Time-on-stream evolution of CO conversion for the Co@SiO_2-*873* and Co/SiO_2 catalysts prepared using conventional methods. '-*MI*' refers to melt infiltration. '-*IWI*' refers to incipient wetness impregnation. '-*A*' refers to Aerosil-200 support and '-*F*' refers to CARiACT Q-10 support. Reaction conditions: 483 K, 20 bar, H_2/CO=1, and syngas flow of 40 ml min^{-1}. (b) Time-on-stream evolution of CO conversion for the Co@SiO_2-*873* and Co/SiO_2-*F-TIWI* catalysts prepared using two-step incipient wetness impregnation method (*TIWI*). Reaction conditions: 483 K, 26 bar, H_2/CO=2, and syngas flow of 40 ml min^{-1}. **(Page 9)**

Table 3 | Catalytic performance of Co@SiO_2-*873* and Co/SiO_2 catalysts prepared using conventional methods.

Sample	Sample weight (mg)	Cobalt loading (wt.%)	X_{CO} (%)	*CTY* (10^{-5} $mol_{CO}g^{-1}_{Co}s^{-1}$)	*S* [%]			
					C1	C2-C4	C5+	CO2
Co@SiO_2-*873*[a]	100	51	15.2	4.2	5.2	3.8	91.0	-
Co/SiO_2-*A-MI*[a]	100	42	7.5	2.6	4.5	4.1	91.5	-
Co/SiO_2-*F-MI*[a]	100	42	8.6	3.0	4.8	4.3	90.9	-
Co/SiO_2-*F-IWI*[a]	250	16.5	8.7	3.1	4.7	4.9	90.4	-
Co@SiO_2-*873*[b]	175	51	70.2	7.8	9.7	5.2	84.7	0.4
			25.8	6.4	10.7	6.5	82.8[c]	-
Co/SiO_2-*F-TIWI*[b]	175	32	19.1	3.5	9.0	7.4	83.6	-
			26.0	3.4	9.3	7.6	83.0[c]	-

Carbon conversion (X_{CO} , %), activity per gram of Co (*CTY*), hydrocarbon selectivity (*S*, %). (a) FTS experiments were carried out at 483 K, 20 bar, and H_2/CO=1, and syngas flow of 40 ml min^{-1}, and data was collected after 201 h *TOS*; (b) FTS experiments were carried out at 483 K, 26 bar, and H_2/CO=2, and syngas flow of 40 ml min^{-1}, and data was collected after 100 h *TOS*. (c) C5+ selectivity was obtained after 118 h *TOS* by changing the feed flow rate after 100 h *TOS*. **(Page 9)**

3.The comparison with other preparation techniques (IWI and MI) is unfair since the Co particle size is not evaluated, when these conventional techniques are used. Furthermore, the widely used and easily applied 2-step impregnation is missing from the conventional techniques. Why the authors choose to leave it outside comparison?

We would like to stress here that one of the most novel aspects of our current work is the fact that the presented synthetic protocol is able to deliver highly loaded Co/SiO_2 catalysts with well distributed cobalt nanoparticles, which, to the best of our knowledge, has not been possible using other conventional methods, including '*IWI*' and '*MI*'.

As per request of the referee, we have also prepared catalysts following a two-step impregnation (TIMI) method. As mentioned above, the Co@SiO_2-*873* catalyst has a cobalt-time-yield of 7.8*10^{-5} $mol_{CO}g^{-1}_{Co}s^{-1}$, at least 2 times higher than the Co/SiO_2 catalyst prepared by the *TIWI* method after 100 h time-on-stream.

We also performed TEM analysis of Co/SiO_2-*F-TIMI* catalyst and added these data in Figure S14 in the supporting information. As it can be observed, this sample displays a large number of aggregates.

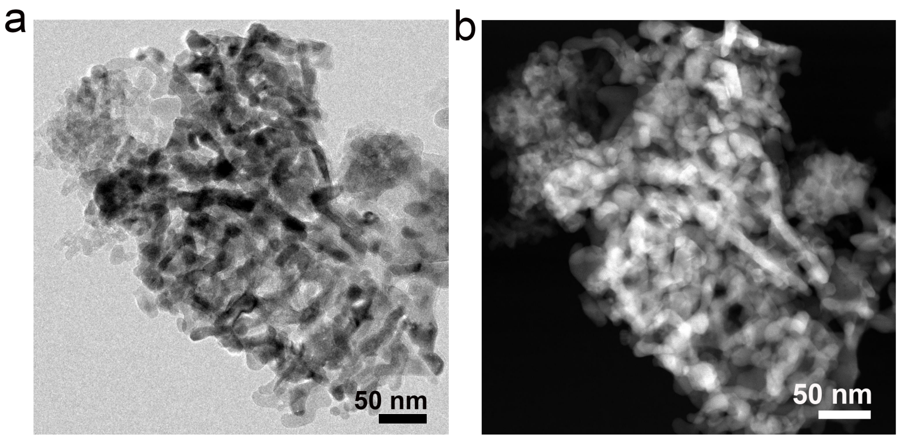

Supplementary Figure 14 Bright field (a) and dark field (b) TEM micrograph of Co/SiO_2-*F-TIMI* catalyst. All scale bars 50 nm. **(Page 12)**

4. Finally the reasoning behind the enhanced performance (1.5 times higher CTY and a=0.94) are not discussed in terms of catalysts properties. Could they be explained simply by the particle size effect? Authors are not validating that the measured particle size with TEM or H2 chemisorption will remain the same after 20 bars of H2. Did the authors performed similar characterization on the spent catalyst?

The reasoning behind the enhanced performance is now discussed in detail in the main article:

<< We argue that the low H_2/CO ratio and operating temperature applied in this work (H_2/CO=1, 483 K) along with an optimal cobalt particle size in the synthesized Co@SiO_2-*873* catalyst result in a chain growth probability (α) as high as 0.94. >> (Page 9)

<< Interestingly, TEM images of the Co@SiO_2-*873* catalyst after 201 h *TOS* show a very good dispersion of cobalt nanoparticles on the SiO_2 support along with very few aggregates (Supplementary Fig. 15a-d). In comparison with other highly loaded catalysts prepared using traditional methods, the optimal particle size and high stability of cobalt nanoparticles in Co@SiO_2-*873* lead to more available cobalt sites and explain the high activity of Co@SiO_2-*873* in the Fischer-Tropsch synthesis process.>> (Page 10)

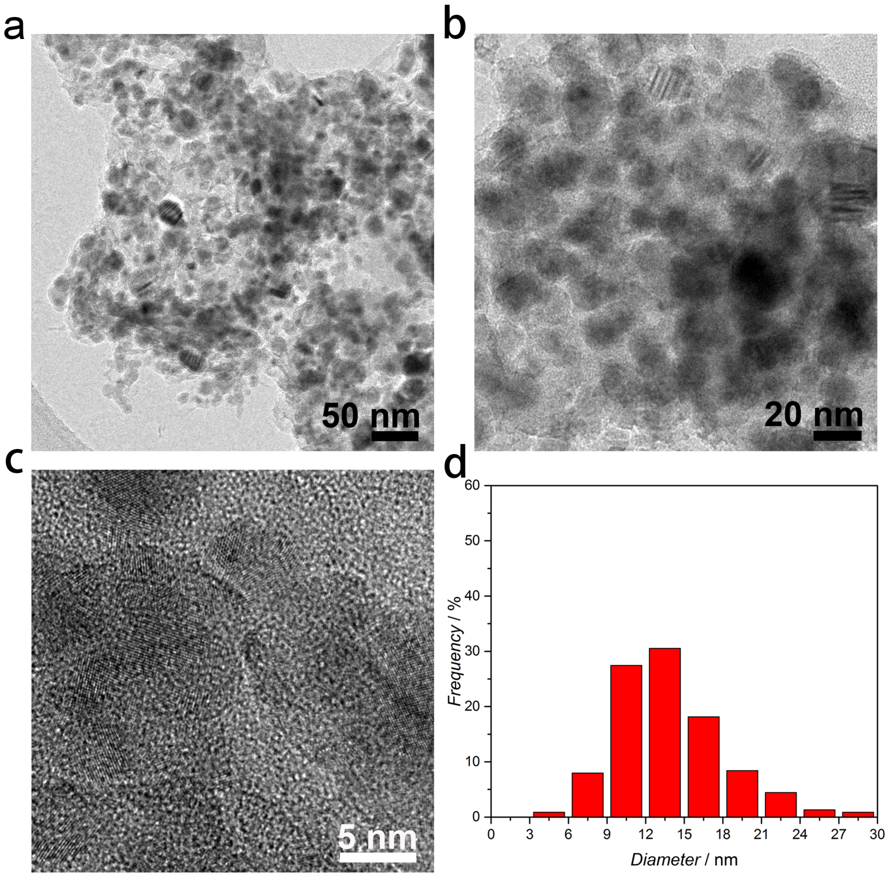

Supplementary Figure 15 Bright field (a,b) and high-resolution (c) TEM micrograph of Co@SiO_2-*873-spent* catalyst. (d) Particle size histograms obtained from TEM analysis for Co@SiO_2-*873-spent* catalyst, with an average particle size of 16 nm. **(Page 13)**

As explained above, we have performed TEM analysis for the spent Co@SiO_2-*873* to obtain more information of the catalyst structure after FT reaction, and added these data in Figure S15 in the supporting information.

Minor comments of consideration:

1: Figure 1 miss the H2 reduction step

The following change has been made:

(4) Reduction of the Co@SiO_2 in H_2 leads to the formation of metallic Co for Fischer-Tropsch synthesis.

2: Since the study refers to the structure-sensitivity of FTS a reference to the first paper discovering the phenomenon Journal of Catalysis 200, (2001) 106 in addition to refs 13 and 14 has to be included.

The suggested reference has been added.

(15) Barbier A, Tuel A, Arcon I, Kodre A, Martin GA. Characterization and Catalytic Behavior of Co/SiO_2 Catalysts: Influence of Dispersion in the Fischer–Tropsch Reaction. Journal of Catalysis 200, 106-116 (2001).

3: Similarly the addition of the first paper describing the formation of needle-like structures of Cobalt and silica on FT catalyst by Exxon Journal of Catalysis 217 (2003) 127.

Added:

(34) Kiss G, Kliewer CE, DeMartin GJ, Culross CC, Baumgartner JE. Hydrothermal deactivation of silica-supported cobalt catalysts in Fischer–Tropsch synthesis. Journal of Catalysis 217, 127-140 (2003).

4: Authors use XRD to claim that carbon is preventing oxidation by air. XRD probes long range order and oxidized Co nuclei of few layers would not be detected. This statement could only be made if in situ XRD would be used. It is surprising that the catalysts used directly after pyrolysis have some activity, since they are fully covered with carbon.

We agree with this comment. At the same time, the accessibility of some cobalt nanoparticles was confirmed by the acid-leaching experiment, as shown in Table 2 in the Supporting information. Some cobalt nanoparticles can be removed in hydrogen chloride solution. Thus, these accessible cobalt nanoparticles can offer some active sites for the FTS reaction.

Based on the suggestions from Reviewer 2, we have modified the text in the main article, as presented below:

<< According to HR-TEM, during pyrolysis, cobalt nanoparticles are encapsulated by multilayers of graphitic-carbon shells (Supplementary Fig. 5g-i) that render them, most likely, inaccessible. XRD analysis further confirm this observation, since re-oxidation of most Co does not occur upon exposure to atmospheric conditions (*vide supra*). In addition, leaching experiment using HCl demonstrates that only a 30% of cobalt can be leached (Supplementary Table 2). >> (Page 4)

Reference

1. Botes FG. Influences of Water and Syngas Partial Pressure on the Kinetics of a Commercial Alumina-Supported Cobalt Fischer−Tropsch Catalyst. *Industrial & Engineering Chemistry Research* **48**, 1859-1865 (2009).

2. Satterfield CN, Hanlon RT, Tung SE, Zou ZM, Papaefthymiou GC. Effect of water on the iron-catalyzed Fischer-Tropsch synthesis. *Industrial & Engineering Chemistry Product Research and Development* **25**, 407-414 (1986).

3. Botes FG, Niemantsverdriet JW, van de Loosdrecht J. A comparison of cobalt and iron based slurry phase Fischer–Tropsch synthesis. *Catalysis Today* **215**, 112-120 (2013).

4. Espinoza RL, Steynberg AP, Jager B, Vosloo AC. Low temperature Fischer–Tropsch synthesis from a Sasol perspective. *Applied Catalysis A: General* **186**, 13-26 (1999).

5. van Steen E, Claeys M, Dry ME, van de Loosdrecht J, Viljoen EL, Visagie JL. Stability of Nanocrystals: Thermodynamic Analysis of Oxidation and Re-reduction of Cobalt in Water/Hydrogen Mixtures. *The Journal of Physical Chemistry B* **109**, 3575-3577 (2005).

6. Ma W*, et al.* Fischer–Tropsch Synthesis: Influence of CO Conversion on Selectivities, H2/CO Usage Ratios, and Catalyst Stability for a Ru Promoted Co/Al2O3 Catalyst Using a Slurry Phase Reactor. *Topics in Catalysis* **54**, 757 (2011).

7. Bukur DB, Pan Z, Ma W, Jacobs G, Davis BH. Effect of CO Conversion on the Product Distribution of a Co/Al2O3 Fischer–Tropsch Synthesis Catalyst Using a Fixed Bed Reactor. *Catalysis Letters* **142**, 1382-1387 (2012).

8. Yang J, Ma W, Chen D, Holmen A, Davis BH. Fischer–Tropsch synthesis: A review of the effect of CO conversion on methane selectivity. *Applied Catalysis A: General* **470**, 250-260 (2014).

9. Bezemer GL*, et al.* Cobalt Particle Size Effects in the Fischer−Tropsch Reaction Studied with Carbon Nanofiber Supported Catalysts. *Journal of the American Chemical Society* **128**, 3956-3964 (2006).

Reviewer #1 (Remarks to the Author)

The manuscript should be retruned to the authors so they can make a much improved response to the reviewer's comments.

For example, in response to Reviewer 1 comments the authors state that "Fe catalysts show almost no activity under low temperature FTS cibdutuibs..." This statement is absolutely not true. Sasol workers clearly show in graphical form where Co catalysts are superior and where Fe catalysts are superior.

Reviewer #2 (Remarks to the Author)

The manuscript by Gascon et al. presents the use of MOF as sacrificial templates for the preparation of cobalt based catalysts for Fischer – Tropsch synthesis. The authors report on the long standing challenge of catalyst preparation at high loadings of the active metal, a topic especially important for unlocking small scale applications of FTS technology. In addition, the manuscript paves the way for new approaches in breaking the interdependence of crucial structural parameters influencing FTS performance. The manuscript is well constructed and written having the potential to be published at Nature communications. However, clearly not all claims are supported by the presented data. Therefore I would recommend publication only after major revision. The major issues leading to this suggestion are summarized in the following points:

Authors claim better activity, selectivity to higher hydrocarbons and stability for the catalysts prepared by the MOF templating method.

1.The claim of excellent stability cannot stand with only the presented data. All tested catalysts appear to have very similar stability (Figure 10, supporting) with the exception of Co@SiO2-cal. catalyst that shows some deactivation. Therefore, it cannot be concluded that the presented preparation procedure outperforms in terms of stability catalytic materials prepared by conventional methods. In addition, and more importantly the tests have been performed at moderate to low carbon monoxide conversions (<20%) and consequently low partial pressures of steam. In Co based FTS it is well accepted that the initial deactivation rate is related to pH2O, consequently tests at more realistic conversions (>50%) would allow a clear statement on stability.

2.The effect of water in FTS is paramount. Apart from catalyst stability, the H2O formed affects both activity and selectivity. Conversion level has a positive influence into the C5+ selectivity therefore comparison of small differences (see Tables 2 and 3) is valid only for data obtained at similar

conversion levels. In addition, a low H_2 to CO ratio is used ($H_2/CO=1$) that is far from the stoichiometric (2.1). This renders comparison with literature values unfair since low ratios are boosting C_{5+} up to 15%. Finally, the carbon removed as CO_2 is not considered in the calculations increasing the error.

3.The comparison with other preparation techniques (IWI and MI) is unfair since the Co particle size is not evaluated, when these conventional techniques are used. Furthermore, the widely used and easily applied 2-step impregnation is missing from the conventional techniques. Why the authors choose to leave it outside comparison?

4.Finally the reasoning behind the enhanced performance (1.5 times higher CTY and a=0.94) are not discussed in terms of catalysts properties. Could they be explained simply by the particle size effect? Authors are not validating that the measured particle size with TEM or H_2 chemisorption will remain the same after 20 bars of H_2. Did the authors performed similar characterization on the spent catalyst?

For the above reasons I believe that the study needs significant rewriting and/or additional experimental work in order to convince on the clear benefit of the suggested preparation route.

Minor comments of consideration

•Figure 1 miss the H_2 reduction step

•Since the study refers to the structure-sensitivity of FTS a reference to the first paper discovering the phenomenon Journal of Catalysis 200, (2001) 106 in addition to refs 13 and 14 has to be included.

•Similarly the addition of the first paper describing the formation of needle-like structures of Cobalt and silica on FT catalyst by Exxon Journal of Catalysis 217 (2003) 127.

•Authors use XRD to claim that carbon is preventing oxidation by air. XRD probes long range order and oxidized Co nuclei of few layers would not be detected. This statement could only be made if in situ XRD would be used. It is surprising that the catalysts used directly after pyrolysis have some activity, since they are fully covered with carbon.
